# Enhanced Performance of Nanocomposite Membranes by an Environmentally Friendly High-Pressure Silanization Method

**DOI:** 10.1021/acsomega.4c10503

**Published:** 2025-03-03

**Authors:** Patience Nnenna Abugu, Hanin Samara, Adrián Rojas, Ewelina Ksepko, Mariusz Nowak, Ximena Valenzuela, Marcin Tyrka, Philip Jaeger, Irena Zizovic

**Affiliations:** † Faculty of Chemistry, Wroclaw University of Science and Technology, Wyb. Wyspianskiego 27, 50-370 Wroclaw, Poland; ‡ Department of Materials Science and Metallurgy, University of Cambridge, 27 Charles Babbage Road, CB3 0FS Cambridge, U.K.; § Institute of Subsurface Energy Systems, Clausthal University of Technology, Agricolastr. 10, 38678 Clausthal-Zellerfeld, Germany; ∥ Packaging Innovation Center (LABEN), Department of Science and Food Technology, Faculty of Technology, University of Santiago of Chile, Obispo Umaña 050, Santiago 9170201, Chile; ⊥ Center for the Development of Nanoscience and Nanotechnology, Santiago 9170124, Chile

## Abstract

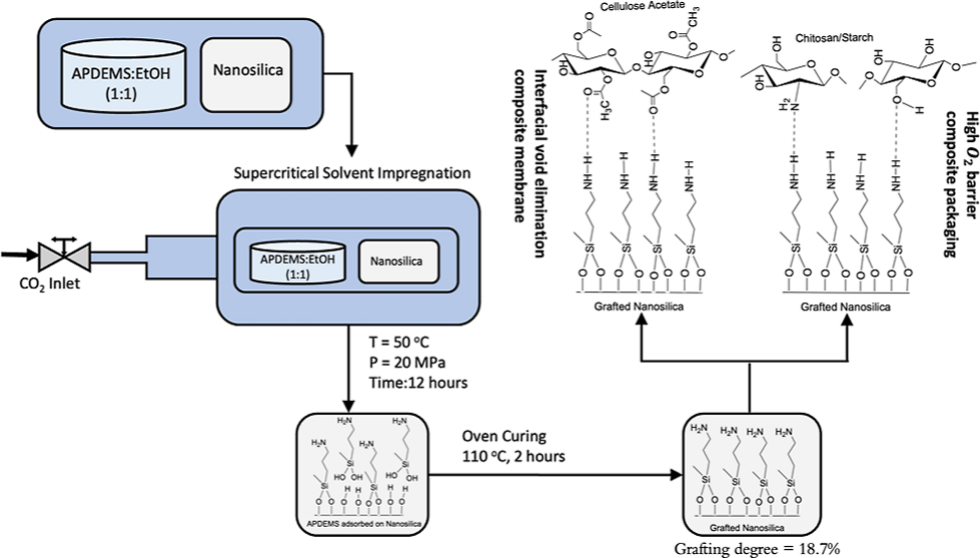

Composite membranes that contain an inorganic phase dispersed in a polymer are valuable for various applications because they combine the flexibility of polymers with the excellent thermal stability and gas selectivity of inorganic materials. The appearance of voids at the interface between the organic and inorganic phases is a central issue in such nanocomposite design. One approach to tackling this issue is grafting the inorganic phase with silane coupling agents that serve as linkers for the two phases. The study reports the structural improvement of nanocomposite membranes by filler silanization in an environmentally friendly process. Silanization of silica nanoparticles with 3-aminopropyldiethoxymethylsilane was carried out by using supercritical carbon dioxide. The process based on supercritical solvent impregnation was designed to minimize the loss of nanoparticles and the generation of organic effluents. Surface-modified nanoparticles were obtained with grafting degrees of up to 18.7%. SEM imaging confirmed less prominent nanoparticle agglomeration after the modification. In the next step, nanocomposite membranes based on cellulose acetate (CA) (20 wt % nanosilica) and starch-chitosan blend (15 and 20 wt % nanosilica) were fabricated using solvent-casting. Dope solutions were prepared using neat and silanized nanosilica by employing an alternation of stirring and sonication. The membranes containing modified nanoparticles showed a more homogeneous structure. The silanization apparently improved the compatibility of the nanosilica with the polymers. Consequently, high-pressure hydrogen permeation through the nanocomposite CA-based membrane with 20 wt % modified nanoparticles was considerably reduced (93%) compared to the membrane with the pristine nanosilica. The oxygen barrier properties of the starch-chitosan blend were improved by 98.3% by adding 20 wt % of modified nanosilica, yielding a film with an oxygen permeability value as low as 0.007 cm^3^ mm/(m^2^ atm day).

## 1 Introduction

Composite membranes comprising polymers and inorganic fillers are of interest for numerous applications, from gas separation to novel food packaging design.^1,2^ For this purpose, surface-modified nanosilica is considered a well-established material for composite membrane design.^3^ Commonly, inorganic fillers need to undergo surface treatment to ensure a homogeneous and stable dispersion and enhanced bonding between the dispersed inorganic and continuous organic phases. Silanization, i.e., organosilane grafting (covalent bonding) to surface hydroxyl groups of nanosilica, is a way to perform the required modification of nanoparticles. It was previously shown that silanization decreases nanoparticle agglomeration, enables better nanoparticle distribution, prevents an undesirable viscosity increase of composite formulations, and reduces nanosilica incompatibility with the organic phase.^3^ The surface-modified nanosilica improved rubber’s durability,^4^ bitumen’s aging resistance,^5^ and scratch resistance of polymer coatings.^6^

Due to its specific properties, supercritical carbon dioxide (scCO_2_) has been increasingly applied in many areas. High density, low viscosity, high diffusion coefficients, and the absence of surface tension in the supercritical phase allow its easy penetration into solid matrices, which opens the possibility for many applications.^7^ ScCO_2_ can act as a solvent, polymer plasticizer, transporter, and reaction medium. An essential advantage of processes involving scCO_2_ is the minimization or complete elimination of effluent and solid waste generation.^8^ Therefore, the first industrial facilities for impregnation using scCO_2_ were built as breakthroughs in highly polluting industries such as wood treatment (Superwood, Hampen, Denmark) and textile dyeing (DyeCoo, Weesp, The Netherlands).

The conventional silanization of inorganic particles includes extensive usage of organic solvents such as toluene or ethanol and stirring at temperatures up to 80 °C for periods ranging between 1 and 24 h.^3,9^ The processing also requires a washing and a liquid–solid separation step. Consequently, organic effluents are produced, and particle loss, in the case of nanosilica, cannot be avoided. As an environmentally friendly alternative to conventional silanization processes, organosilane grafting in scCO_2_ emerged. Silanization in the presence of scCO_2_ was reported as a feasible process for cellulose,^10^ cellulose acetate,^11^ and silica^12,13^ as base materials. To the best of our knowledge, there are only three studies dealing with nanosilica coating with silane in scCO_2_, all of which pertain to methacryloxypropyltrimethoxy (MEMO) silane and the preparation of PMMA-based composites^14−16^ with 1–7 wt % of nanosilica. The coating was performed either by nanoparticle mixing in the supercritical phase^14−16^ or using a packed bed with glass beads,^15^ which are laboratory-scale processes. Therefore, it could be concluded that despite the advantages of using supercritical fluids, nanosilica modification with silanes in scCO_2_ for composite design has been poorly explored from the point of processing methods, the choice of organosilanes and polymers used, and potential applications of prepared composites. Composite membranes with inorganic fillers are of particular interest in gas separations. Mixed matrix membranes (MMMs) contain an inorganic phase dispersed in a polymer and have the potential to achieve higher selectivity, permeability, or both relative to the existing polymeric membranes,^17^ as they combine the benefits of the flexibility of polymers and the excellent selectivity of inorganic materials. Silica has been investigated as a filler in MMM preparation, and most reports are summarized in the review of Setiawan and Chiang.^18^ It was shown that the CO_2_/N_2_ selectivity of cellulose acetate/nanosilica composite membranes prepared by the thermal-inversion method increased by adding 20 wt % of silica.^19^ Another study^20^ showed increased permeation of O_2_ and CH_4_ in polysulfone MMMs with up to 20 vol % of fumed nanosilica obtained by the solvent casting method. However, the incorporation of nanosilica did not result in an overall improvement in the performance of permeability versus selectivity.^20^ Still, data on the application of surface-modified silica in composite membrane design are scarce and relate only to mesoporous silica particles. Wu et al.^21^ showed increased gas permeability and CO_2_/CH_4_ and CO_2_/N_2_ selectivities in composite membranes with poly(ether-*block*-amide) and 20 wt % amine-functionalized mesoporous silica MCM-41 obtained by the solvent casting method. To the best of our knowledge, there are no reports in the open literature on using aminosilane-modified nanosilica and scCO_2_-silanized inorganic particles of any kind in nanocomposite design aimed at gas separation.

Research has shown that nanocomposites can enhance food packaging by improving its mechanical, thermal, and barrier properties.^22−24^ It was also reported that the nanoclay addition to the polymeric phase might modify the release rate of an active component from active food packaging.^25^ While many nanomaterials have been investigated, such as nanoparticles of silver, TiO_2_, ZnO, MgO, silica, and single- and multiwalled carbon nanotubes (MWCNTs), polymer composites with nanoclays and montmorillonite remain the most studied.^22,23,25^ Common filler contents used to design food packaging range from 1 to 7 wt %.^22,23^ From a recent review,^22^ it can be concluded that several reports deal with nanosilica, whereby its content was up to 5 wt %.

**Figure 1:**
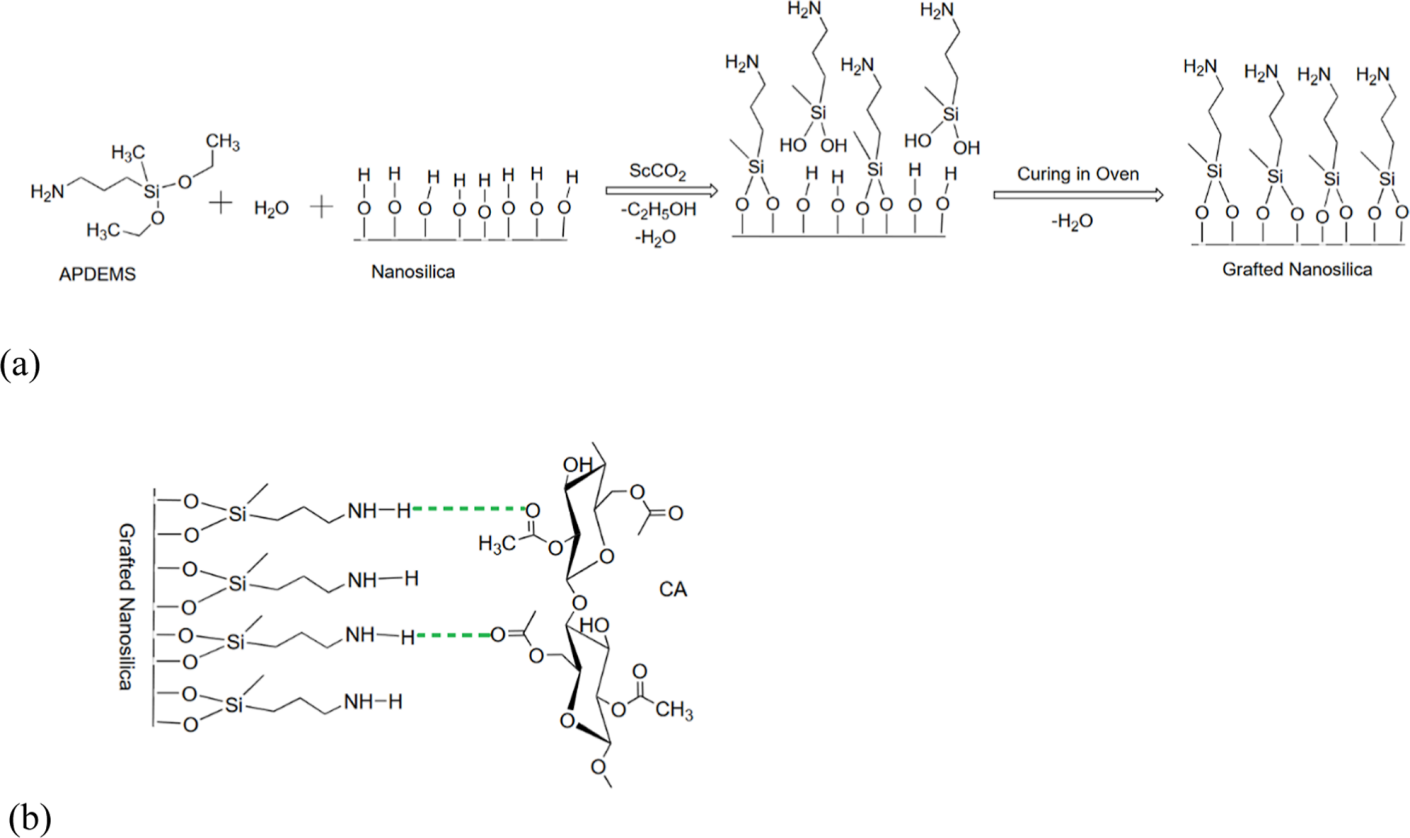
Reaction between the surface hydroxyl groups of nanosilica and APDEMS (a), and an example of the expected interface improvement with cellulose acetate (CA) due to silanization–hydrogen bonding interactions shown in green (b).

The first part of this study explores the environmentally friendly supercritical silanization of nanosilica in a way that can offer early data for scale-up. Considering the easy penetration of scCO_2_ through solids due to the absence of surface tension, the silanization reaction (Figure 1a) was investigated for tightly packed nanosilica particles. In the next step, the expected improvement in compatibility with the organic phase (Figure 1b) is tested for two different composite systems within two case studies. The first case study investigates the potential use of surface-modified nanosilica in preparing cellulose acetate-based nanocomposite membranes aimed at gas separation, while the second case study pertains to preparing composite membranes with a starch-chitosan (SC) blend, envisaged to design packaging material with increased oxygen barrier properties. The central issue in both applications is the prevention of void formation at the interface between the organic and inorganic phases. This is especially important in composites with high nanosilica content requirements (e.g., 15 wt % and higher), such as in nanocomposite membranes for gas separation.

## 2 Experimental Section

### 2.1 Materials

Spherical and porous silicon dioxide nanoparticles (5–20 nm) and 3-aminopropyldiethoxymethylsilane (APDEMS, 97%) were purchased from Sigma-Aldrich (Germany). Ethanol (99.9%) was provided by Stanlab (Poland), and carbon dioxide (purity ≥99.5%) was supplied by SIAD (Poland). Food grade potato starch (PPZ Trzemeszno, Poland) bought at the local market, chitosan (high molecular weight, 75% deacetylated) supplied by Sigma-Aldrich, acetic acid (99.5–99.9%) and anhydrous glycerol provided by POCH (Poland) were used to produce SC blend membranes. Cellulose acetate powder (Mn ∼ 30 000, 39.8% acetyl cont.) purchased from Sigma-Aldrich and acetone (p.a.) supplied by Stanlab were used for cellulose acetate-based nanocomposite preparation.

### 2.2 Silanization in a Supercritical Phase

**Figure 2:**
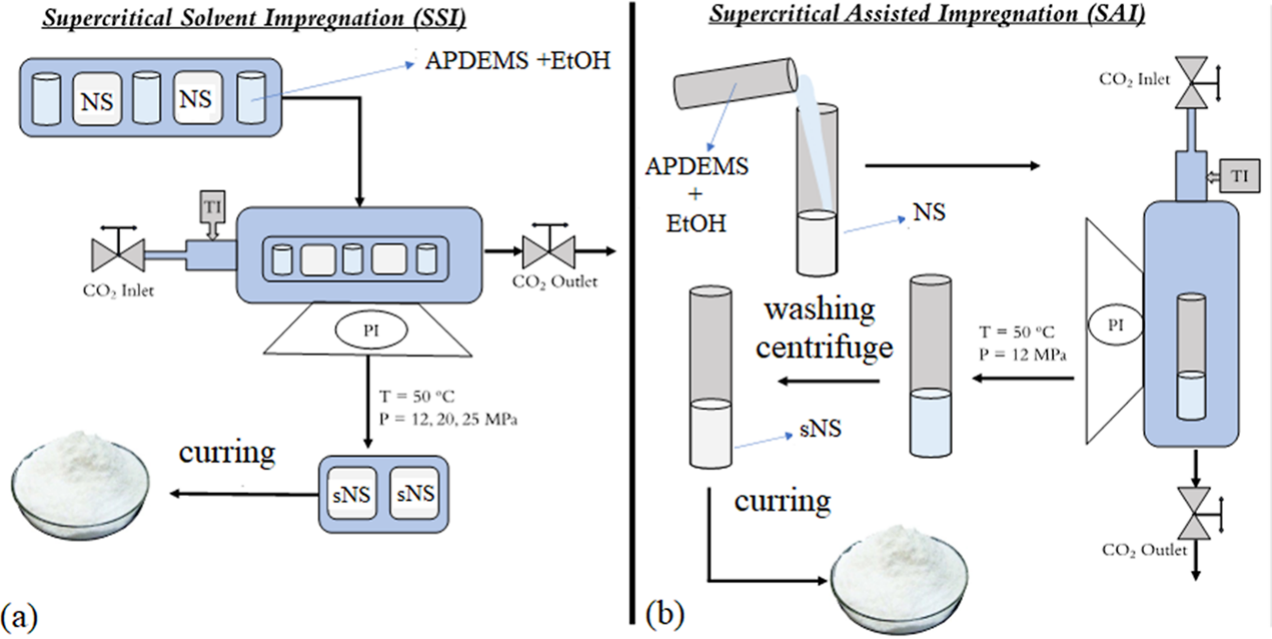
Illustration of SSI (a) and supercritical assisted impregnation (SAI) (b).

**Figure 3:**
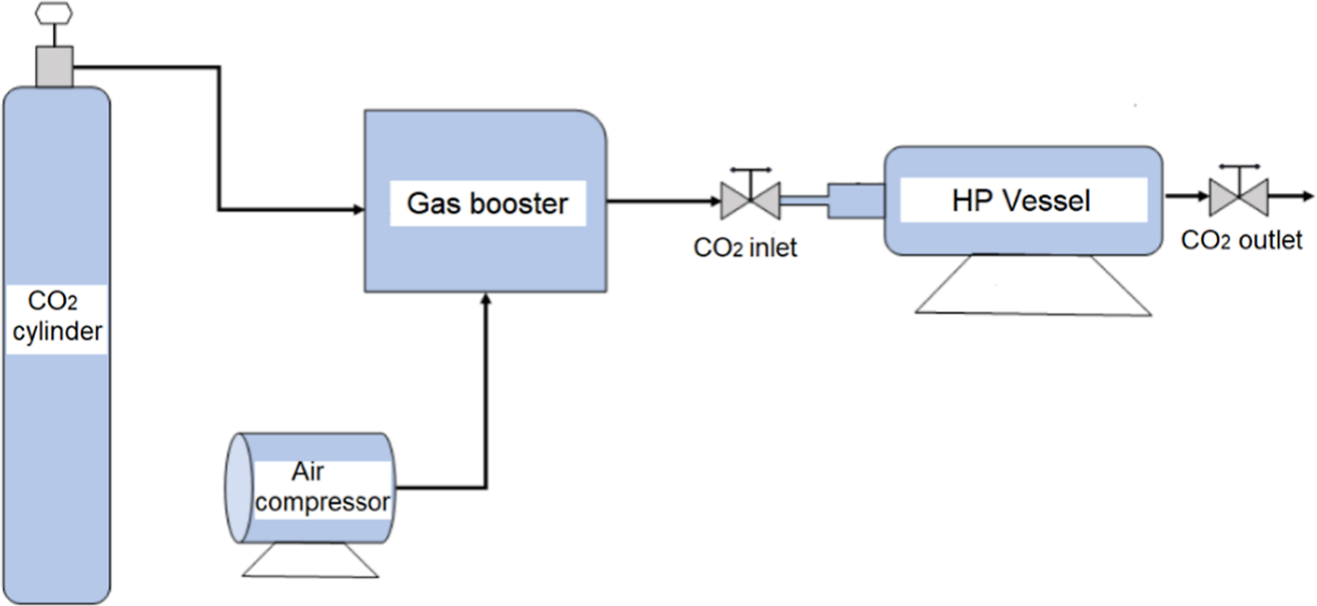
Experimental setup for nanosilica grafting with aminosilane in scCO_2_.

The silanization reaction of nanosilica with APDEMS (Figure 1a) was performed in a 280 mL tiltable high-pressure vessel (*P*_max_ = 50 MPa, *T*_max_ = 120 °C, Eurotechnica GmbH, Germany) (Figure S1, Supporting Information). Two high-pressure techniques were employed, and their simplified presentations are presented in Figure 2. The whole experimental setup, including the high-pressure compressor, is shown in Figure 3. The first technique employed was supercritical solvent impregnation (SSI) (Figure 2a). In this process, nanoparticles and aminosilane (APDEMS) solutions do not come into direct contact with one another. Instead, scCO_2_ was used as a solvent for APDEMS and its transport medium to nanosilica. In a typical experiment, a mass of 0.60 g of nanosilica was divided into two glass containers and wrapped in filter bags to prevent particle loss (Figure S1, Supporting Information). A volume of 8.0 mL of a 1:1 vol mixture of aminosilane and ethanol was placed in smaller glass vessels surrounding the containers with nanosilica arranged in a metal holder (Figure S1). The desired temperature of the autoclave was achieved via a heating jacket and water as a heating medium. In the next step, CO_2_ was introduced into the high-pressure vessel and compressed using an air-driven gas booster (*P*_max_ = 35 MPa, Maximator GmbH, Germany) (Figure 3). The reaction was carried out at a constant temperature of 50 °C based on previous studies on silanization in scCO_2_,^10,11^ while the pressure (12, 20, and 25 MPa) and impregnation time (4, 8, and 12 h) were varied. After the impregnation reaction, the system was decompressed at an average rate of 0.5 MPa/min, and the resulting dry product was taken for curing at 110 °C for 2 h.

The silanization reaction was also performed using supercritical assisted impregnation (SAI) as the second high-pressure method for comparison (Figure 2b). Unlike SSI, silica nanoparticles were in direct contact with an aminosilane solution during this process. The main role of scCO_2_ is the intensification of the contact between the solid and liquid phases, and therefore, SAI is usually carried out at lower pressures than SSI. A mass of 0.60 g of nanosilica was stirred in 8.0 mL of a 1:1 vol solution of aminosilane in ethanol, and the mixture was put in the tiltable high-pressure vessel placed in a vertical position. Afterward, CO_2_ was introduced into the vessel, and the impregnation reaction was allowed to proceed at 50 °C and 12 MPa for 1 h. The decompression was performed at an average rate of 0.5 MPa/min, and the final product was washed and centrifuged twice in ethanol at 3600 rpm for 10 min. The washed product was then cured in the oven at 110 °C for 2 h for complete water removal.

#### 2.2.1 APDEMS Solubility in scCO_2_

The solubility data is necessary for the process design and understanding of the phenomena occurring during the SSI. The solubility of APDEMS in scCO_2_ under 50 °C and pressures of 12, 20, and 25 MPa was determined by a static method described in our previous study^26^ using a high-pressure view cell (Eurotechnica GmbH, Germany; 25 mL volume, *P*_max_ = 35 MPa, *T*_max_ = 120 °C). A glass container with around 0.8 g of APDEMS was placed in a high-pressure view cell. A stainless steel net was placed on top of the glass container to minimize the APDEMS precipitation during the decompression. The cell was heated by using an electrical heating jacket and then pressurized by the high pressure gas booster. The system was left under the predetermined conditions for 24 h and decompressed at a rate of 0.5 MPa/min. The solubility was calculated from the APDEMS mass difference in the glass container before and after the exposure to scCO_2_, using an analytical balance with an accuracy of ±0.00001 g.

### 2.3 Composite Membranes Preparation

Composite membranes were prepared by using the solvent casting method. The preparation procedure was optimized in preliminary experiments by applying different sequences and durations of sonication, mixing, and priming and adjusting the drying conditions. The filler was kept in an oven at 110 °C for an hour before membrane preparation. To prepare cellulose acetate nanocomposite membranes, 15 mL of acetone was added to 0.25 g of pristine or grafted silica nanoparticles and mixed for 2 h. The particles’ mass (0.25 g) was selected to yield a nanocomposite membrane with 20 wt % of the filler content, a value previously reported as favorable filler content in MMMs for gas separation.^18−21^ After being stirred, the mixture was sonicated for 15 min. In the next step, 1 g of cellulose acetate powder was added to the solution stepwise, and the mixture was stirred for another 2 h. Afterward, sonication for 15 min, stirring for 30 min, and sonication for 30 min were performed in the previously mentioned order. Finally, the solution was poured into a Petri dish (9 cm in diameter) and dried at 50 °C overnight in an oven. The preliminary research revealed that drying temperatures ranging from 20 °C (room temperature) to 50 °C yielded composite membranes of a uniform structure. Temperatures higher than 60 °C induced fast solvent (acetone) evaporation and the formation of streams of evaporating solvent that led to less homogeneous composite structure and sometimes crack formation (observed at 70 °C). The drying temperature of 50 °C was selected for further study. Pure cellulose acetate membranes were prepared in the same manner (without the filler).

To produce SC blend membranes, a chitosan solution was prepared by dissolving 1.0 g of chitosan in 100 mL of 2% (w/v) acetic acid in distilled water. Separately, 1.0 g of potato starch was dissolved in 100 mL of cold distilled water, which was then heated to 90 °C to allow for the formation of thermoplastic starch via starch gelatinization. Afterward, the chitosan and starch solutions were mixed, and glycerol (25 wt % of total solid polymer) was added to the mixture as a plasticizer. The resulting polymer blend solution was allowed to cool to room temperature, degasified in an ultrasonic bath, and then cast into polypropylene molds to achieve a coverage of around 0.51 cm^3^ of the solution per cm^2^ of mold (optimum coverage determined in preliminary experiments). Drying was carried out in an oven at 70 °C for 24 h to obtain SC blend films.

The composite SC blend membranes were prepared with 15 and 20 wt % of pristine and silanized silica nanoparticles with respect to the final nanocomposite membrane. The filler content was selected based on the primary role of the packaging, which was enhancing the oxygen barrier properties. Therefore, the filler contents similar to those in membranes for gas separation were chosen.^18−21^ The nanoparticles were stirred with 40 mL of distilled water for 15 min and then sonicated for another 15 min. In the next step, 40 mL of the previously prepared SC polymer solution (as previously described) was added to the suspended nanoparticles, and the resulting mixture was stirred for 15 min, followed by sonication for another 15 min. Subsequently, the remaining part of the SC blend solution was added while stirring. The mixture was stirred for another 15 min and then deaerated for 30 min during the sonication process. The resulting mixture was poured into polypropylene molds (approximately 0.51 cm^3^/cm^2^) and left in an oven at 70 °C for 24 h. Drying temperatures below 70 °C, required a long drying time, while temperatures of 90 °C and higher yielded wrinkled and nonhomogeneous membranes. The composites dried at 70 and 80 °C showed good uniformity and retained their shape (mold). Accordingly, a drying temperature of 70 °C was selected for the study.

### 2.4 Materials Characterization

Fourier transform infrared (FTIR) spectroscopy was applied to pristine and grafted silica nanoparticles to verify the silanization reaction. The structural properties of the composite membranes were investigated by a two-beam microscope SEM/Ga-FIB FEI Helios NanoLab TM 600i (FEI, Thermo Fisher Scientific, Eindhoven, The Netherlands). Thermogravimetric analysis (TGA) was employed to determine the grafting degree (GD) in modified nanoparticles using a STA 449 F5 Jupiter thermal analyzer coupled to a QMS 403 Aolos Quadro quadrupole mass spectrometer (Netzsch, Germany). The GD was calculated using the following equation^15,27^
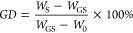
1where GD is the percentage of APDEMS bound to the particles’ surface (GD), *W*_S_ is the remaining weight of pure powder at 800 °C, *W*_0_ is the remaining weight of APDEMS at 800 °C, and *W*_GS_ is the remaining weight of the grafted sample at 800 °C.

Sorption of CO_2_ at 273 K using a micromeritics accelerated surface area and porosimetry system (ASAP) 2020 was used to investigate nanopowder surface areas before and after silanization. This method was selected because the device detects pores in the range from 0.4 to 1.5 nm, which is appropriate considering the size of nanoparticles (5–20 nm). For the differential scanning calorimetry (DSC) testing of SC blend membranes, a Mettler-Toledo model STAR 822e (Schwerzenbach, Switzerland) device coupled with a HAAKE EK 90/MT (Newington, USA) cooling system was used. The details of all analyses applied are provided in the Supporting Information (Section S2.4).

### 2.5 High-Pressure Permeation Tests

**Figure 4:**
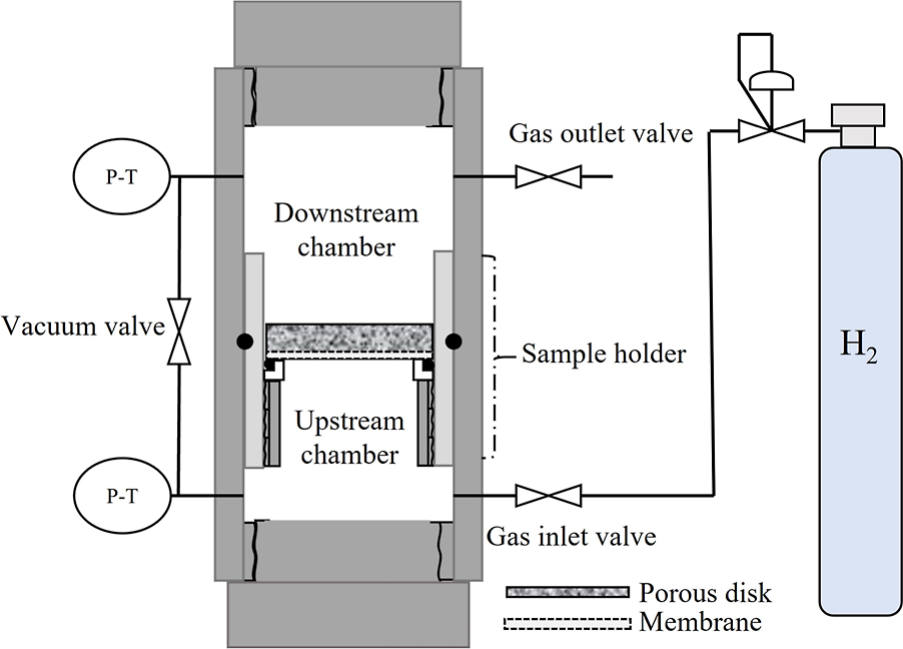
Schematic diagram of the high-pressure permeation setup.

Permeation tests with cellulose acetate-based composite membranes were carried out in a custom-built gas permeation cell manufactured at Clausthal University of Technology (Figure 4) with a maximum operating pressure and temperature of 35 MPa and 120 °C, respectively. The permeation cell consists of one compartment (*V* = 40 mL) partitioned into an upstream chamber and a downstream chamber of precisely known volumes by means of a sample holder. Prior to experiments conducted using the membranes, the proper partitioning of the two chambers and the tightness of the sample holder toward the gas are verified by performing a test where a stainless steel disk was placed inside the sample holder and monitoring the pressure for 24 h. The procedure for determining the permeability of the membranes is then initiated. The membranes are cut into circle-shaped films with a radius of 19.8 mm and placed in the sample holder, which comprises a highly porous filter disk that serves as a membrane support. The gas supply is connected to the upstream chamber through a pressure regulator to control the inlet pressure. A vacuum pump is connected to the outlet valve. Vacuum is applied for a minimum of 4 h to the cell and gas inlet lines to eliminate the presence of air and remove any preabsorbed moisture from the membranes. The test is initiated by opening the gas inlet valve and establishing a constant pressure of 1.9 MPa in the upstream chamber. Pressures and temperatures are continuously recorded by using data acquisition software. The tests are conducted at room temperature (20 °C). The steady state permeability (*P*) is calculated through eq 2 when the change of pressure over time is constant, where *l* is the thickness of the membrane (m), Δ*p* is the transmembrane pressure (Pa), and *n* is the molar flux (mol/(m^2^ .s)), as calculated with the GERG equation of state.^28^ The measured permeability is reported in Barrer. For each membrane of a specific composition, at least two permeability tests were conducted
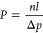
2

## 3 Results and Discussion

### 3.1 Nanosilica Silanization in Supercritical Carbon Dioxide

The silanization outcome was analyzed by thermogravimetry. The residues of the grafted samples detected by TGA (*W*_GS_) and corresponding GDs calculated using eq 1 are presented in Table 1. The observed residue masses of pure nanosilica and APDEMS were 94.16% and 4.46%, respectively. The TGA curves are shown in Figures S2–S7 (Supporting Information). As described in Section 2.2, two techniques (SSI and SAI) were employed. The solubility measurements revealed APDEMS solubility values in scCO_2_ of 0.00485 ± 0.00025, 0.00785 ± 0.00082, and 0.00926 ± 0.0010 g_APDEMS_/g_CO_2__, at 12, 20, and 25 MPa, respectively. The SSI tests performed for 4 h at variable pressures showed poor grafting, with no conversion detected at 12 MPa. Therefore, the processing time was increased to 8 h, and pressures of 20 and 25 MPa were applied to ensure higher APDEMS concentrations in the supercritical phase. As shown in Table 1, the pressure of 20 MPa was more favorable for the grafting reaction, allowing for a higher GD (6.37%) compared to the one obtained at 25 MPa (3.29%). This phenomenon can be explained by a larger affinity of APDEMS toward scCO_2_ at a higher pressure (25 MPa, solubility 0.00926 ± 0.0010 g_APDEMS_/g_CO_2__), consequently leading to its disadvantageous partitioning among the contacting phases and less prominent nanosilica impregnation with silane molecules. Hence, a pressure of 20 MPa was selected as optimal, and the processing time was increased to 12 h. The time increase yielded a significant increase in the GD (18.70%), which can be considered a high value.^29^

**Table 1:** TGA Results and Grafting Degrees as a Function of the Process Parameters at 50 °C

sample	technique	pressure (MPa)	time (h)	*W*_GS_ (%)	GD (%)
1	SSI	20	8	88.79	6.37
2	SSI	25	8	91.30	3.29
3	SSI	20	12	80.03	18.70
4	SAI	12	1	90.85	3.83

The SAI process is characterized by lower pressure and shorter time (e.g., the GD obtained after 1 h at 12 MPa is comparable to that obtained by SSI at 25 MPa for 8 h, as shown in Table 1). However, the processing procedure is more complicated, demanding excessive use of the organic solvent and generating a loss of nanoparticles.

**Figure 5:**
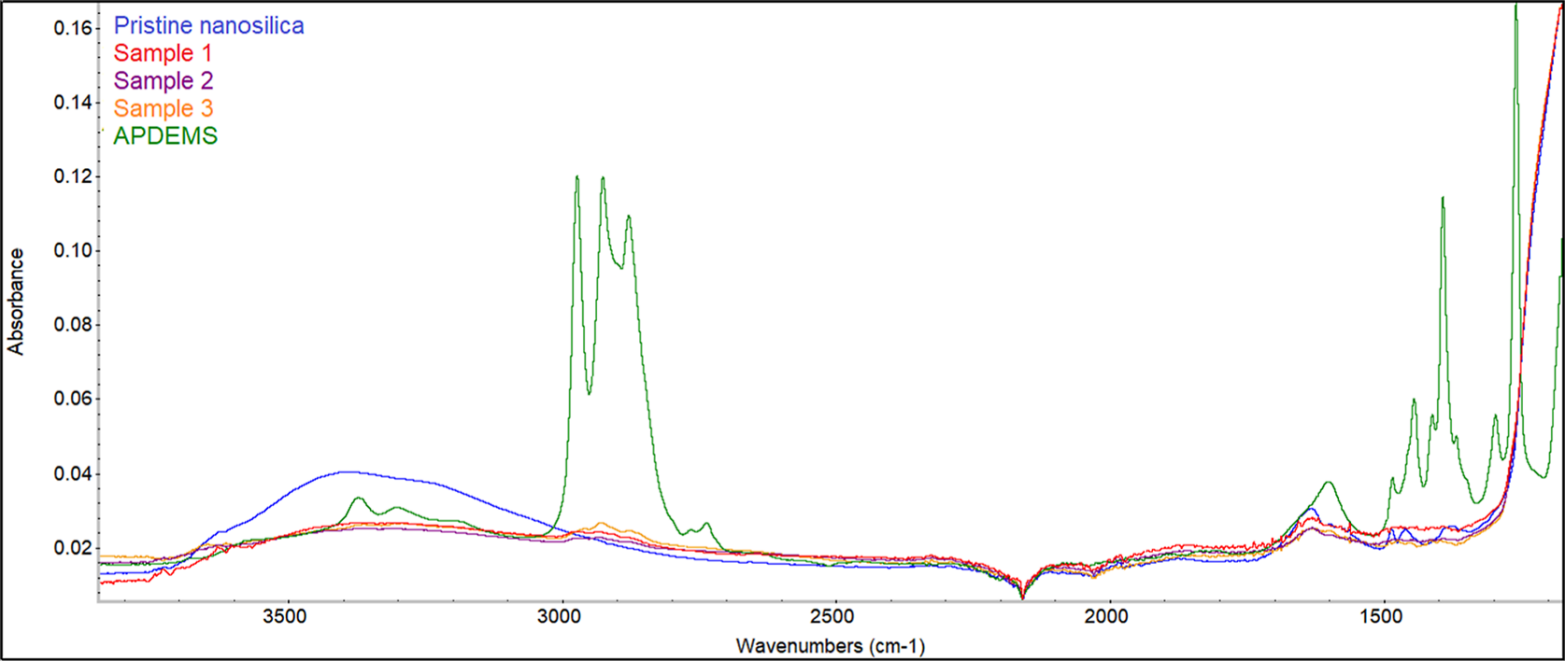
FTIR spectra of pristine nanosilica, modified nanoparticles, and APDEMS.

To confirm the covalent bonding of aminosilane molecules on the surface of nanosilica, we investigated the FTIR spectra of pristine and modified nanosilica samples. Figure 5 shows the FTIR spectra of pristine nanosilica, grafted samples by the SSI (samples 1–3), and APDEMS in the wavenumber range 1300–4000 cm^–1^. Spectra of the full range (wavenumbers from 500 to 4000 cm^–1^) are given in Figure S8 (Supporting Information).

Apart from characteristic signals from symmetric and asymmetric stretching of Si–O–Si bonds in the fingerprint region (below 1000 cm^–1^), the spectrum of pristine nanosilica features a very broad absorption peak centered around 3385 cm^–1^ and another at 1630 cm^–1^, which correspond to stretching of the O–H bond of silanol groups (Si–OH) and adsorbed water (Si–H_2_O) on the surface of nanosilica, respectively.^30,31^ The strongest evidence of covalent bonding between nanosilica and APDEMS is given by the decrease in intensity of this very broad O–H peak of silanol groups, as observed in the spectra of modified nanosilica. It should be noted that the signal at 1630 cm^–1^ (Si–H_2_O), is unaffected by the curing conditions employed because nanosilica particles have been found to retain moisture (physically adsorbed water) after drying at 110 °C.^32^

In the spectrum of APDEMS, the prominent peaks appearing at 2972, 2924, and 2875 cm^–1^ can be attributed to C–H stretching vibrations of CH_3_, CH_2_, and CH groups, respectively, while the stretching and bending vibrations of N–H of the primary amine group occur at 3378 and 1600 cm^–1^, respectively.^1,33,34^ The spectra of all APDEMS-modified nanosilica samples (1–3) present the following features that confirm successful covalent bonding between nanosilica and APDEMS: (i) a significant decrease in the intensity of the broad O–H peak around 3385 cm^–1^ (this peak does not completely disappear because of the APDEMS primary amine N–H stretching around 3378 cm^–1^), (ii) new signals of C–H stretching (CH_3_, CH_2_, and CH) appearing between 2800 and 3000 cm^–1^, and (iii) broadening of the signal around 1630 cm^–1^ due to overlap with N–H bending signal around 1600 cm^–1^ from covalently grafted APDEMS molecule. Also, the intensity of the signal from the C–H stretching of the alkyl group increases with an increase in the GD, and sample 3 (GD = 18.7%) presents the highest intensity.

Sánchez-Vicente et al.^13^ performed surface modification of mesoporous silica SBA-15 with *N*,*N*-(dimethylaminopropyl)trimethoxysilane for carbon capture in an experimental setup similar to ours at 323 K, pressures in the range from 10 to 20.5 MPa for 0.5 to 12 h, and obtained GDs (by TGA) ranging from 5 to 18 wt %, similar to our study. Stojanovic et al.^15^ reported percentages of coated MEMO silane as high as 46.6–80 wt % (by TGA) in experiments by stirring nanosilica particles in the supercritical phase with ethanol at 313 K and pressures 16–20 MPa. This high value is probably due to the formation of vertical multilayers of silanes at the surface. The tendency of triethoxy or trimethoxy silanes to react among themselves and build vertical multilayers is well-known.^35^

SEM images of pristine and grafted (sample 3, GD = 18.7%) nanosilica powders are presented in Figure S9 (Supporting Information). The grafted particles showed a considerably smaller agglomeration. This phenomenon can be attributed to the decreased particles’ surface energy due to silanization.^3^ Based on the presented findings, nanosilica grafted by the SSI at 20 MPa for 12 h (sample 3) was selected for further studies and nanocomposite preparation with cellulose acetate and a SC blend.

The results of low-pressure CO_2_ sorption using Micromeritics ASAP 2020 are presented in Table 2. The powders of pristine nanosilica and silanized nanoparticles with GDs of 6.37% (sample 1) and 18.7% (sample 3) were analyzed. According to the results, the modification has not influenced the surface area considerably. The value obtained for sample 1 was slightly lower than for the pristine powder, while sample 3 exhibited a slightly higher value than the pristine. However, a decrease in micropore volume (*V*_0_) was observed in grafted samples, which is in accordance with the adsorption isotherms (Figure S10, Supporting Information), consequently leading to a smaller calculated mean pore size (*L*_0_). On the other hand, the total pore volume (*V*_DFT_) calculated for slit pores remained slightly lower upon the modification. However, the nature of the pores in the pristine nanopowder is unknown, and this result should be considered with precaution. To the best of our knowledge, there is no similar report in the literature for comparison.

**Table 2:** Surface Area and Porosimetry Analysis for Nanosilica Pores in the Range from 0.4 to 1.5 nm^a^

sample	*S*_0_ (m^2^/g)	*V*_0_ (cm^3^/g)	*E*_0_ (kJ/mol)	*L*_0_ (nm)	*V*_DFT_ (cm^3^/g)
pristine nanosilica	236	0.172	18.80	1.46	0.047
1* (GD = 6.37%)	219	0.079	26.33	0.72	0.044
3* (GD = 18.7%)	255	0.083	28.00	0.65	0.044

a *V*_0_—Dubinin-Raduskevich micropore volume; *S*_0_—Dubinin-Raduskevich specific surface area (cylindrical pores); *L*_0_—mean micropore size by Stoeckly =10.8/(*E*_0_ – 11.4);^36^*E*_0_—characteristic energy of adsorption (Dubinin); *V*_DFT_—DFT total pore volume (slit pores, adsorption branch). *—sample numbers as in Table 1.

The results obtained in this study demonstrate the SSI feasibility for functionalizing tightly packed nanosilica with APDEMS, which is the first step toward process scale-up. The scale-up of SSI is known,^8^ and industrial facilities exist in the textile^37^ and wood^38^ industry sectors as well as medical device development.^39^ The identified conditions for nanosilica silanization (50 °C and 20 MPa) belong to the region of common SSI conditions with pressures between 10 and 30 MPa.^40^ The results imply that the grafting process is poor under lower pressure (12 MPa), indicating the effect of lower APDEMS solubility (0.00485 ± 0.00025 g_APDEMS_/g_CO_2__) compared to conditions of 20 and 25 MPa (0.00785 ± 0.00082 and 0.00926 ± 0.0010 g_APDEMS_/g_CO_2__, respectively). A pressure of 25 MPa yielded less favorable APDEMS partitioning between the solid and supercritical phases due to its large affinity toward scCO_2_. Further, our results identify a time of 12 h as favorable, whereby an increased grafting rate was observed between the 8th and 12th hour of processing at 20 MPa (Table 1). The time of 12 h is acceptable as a starting point for further scale-up studies. For example, the SSI of hip and knee endoprostheses with α-tocopherol requires an impregnation time of 14 h and longer to achieve an even distribution of the active substance in the polymer.^40^ The contact time required at a larger scale can be optimized by employing scCO_2_ forced convection, which would enhance the rates of APDEMS dissolution and mass transfer in the supercritical phase. Forced convection can be achieved by circulating scCO_2_ through the high-pressure vessel utilizing an external pump (e.g., gear pump) or using a magnetic stirrer to induce scCO_2_ convection within the vessel. The external circulation via a pump would require good piping insulation or heating to prevent a temperature drop. In our research, the next step will be to investigate the impact of scCO_2_ convection induced by a magnetic stirrer in a 1 L high-pressure vessel on a tightly packed nanosilica functionalization with APDEMS.

### 3.2 Cellulose Acetate-Based Nanocomposite Membranes

**Figure 6:**
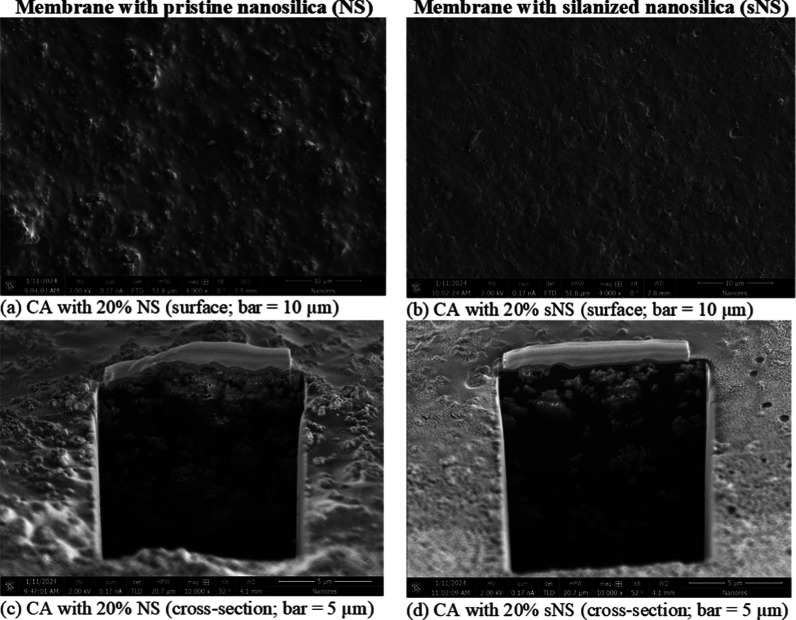
SEM images of CA nanocomposite membranes with pristine nanosilica (a,c) and with silanized nanosilica (b,d) as fillers (CA—cellulose acetate; NS—pristine nanosilica; sNS—silanized nanosilica).

SEM images of surfaces and cross-sections of membranes with pristine and grafted nanosilica are presented in Figure 6. SEM images of the compact structure of the pure cellulose acetate membrane are presented in Figure S11 (Supporting Information). As shown in Figure 6, using APDEMS-grafted filler yielded a smoother membrane surface compared to pristine nanosilica, where several micrometers-large agglomerates were visible. This result is consistent with the considerable agglomeration observed in pristine nanosilica powder (Figure S9, Supporting Information). SEM micrographs of membranes’ cross-sections are presented in Figures 6c,d. Large nanosilica agglomerates were visible in the cross-section of the membrane with the pristine filler (Figure 6c). Figure 6d shows considerably smaller aggregates in the membrane with the grafted filler. Therefore, the silanization decreased the particle agglomeration in the cellulose acetate-based nanocomposite membrane and allowed for better nanoparticle distribution within the composite.

#### 3.2.1 Gas Permeability Tests

**Figure 7:**
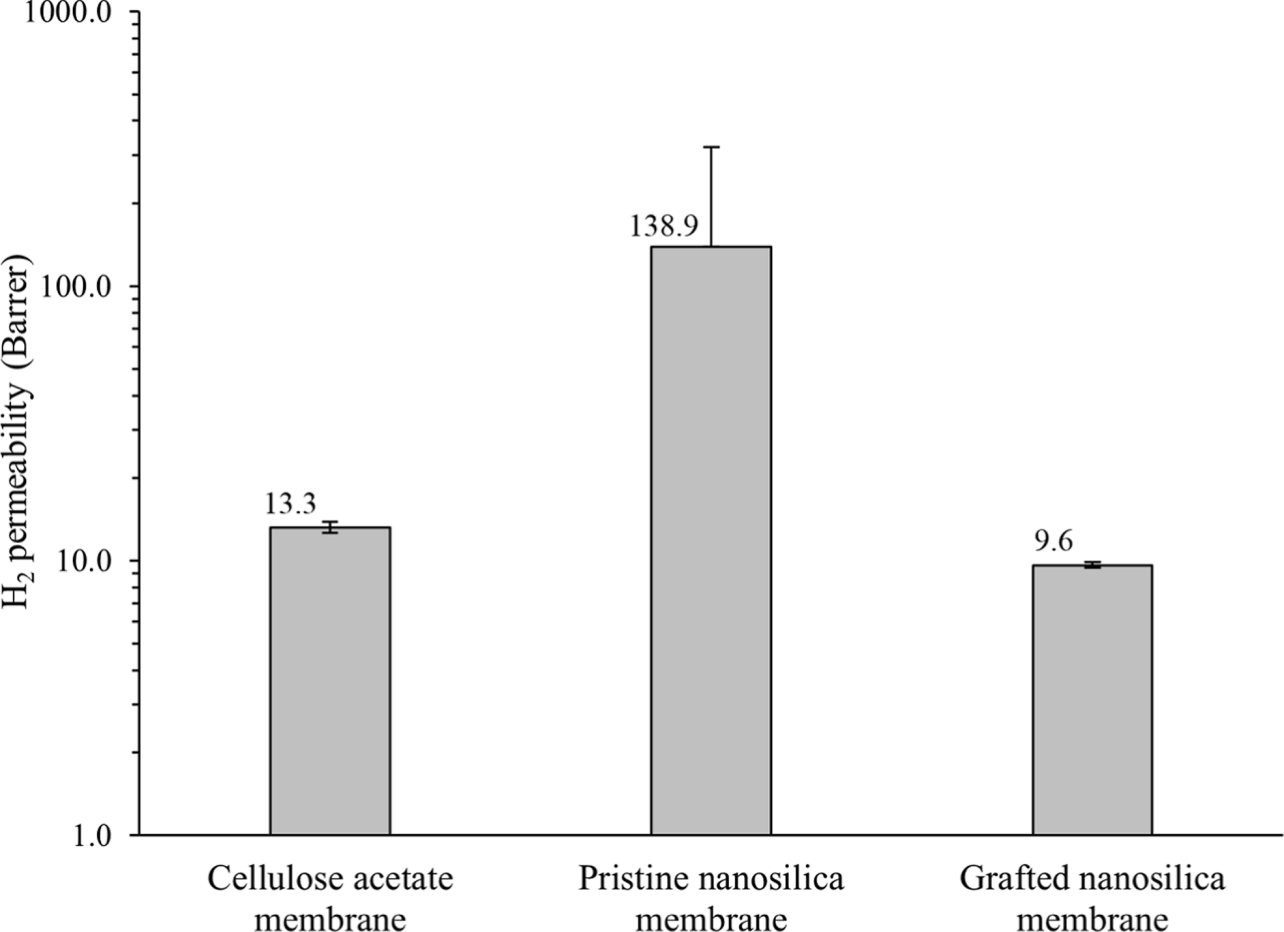
Hydrogen permeability of CA and CA-based nanocomposite membranes with pristine and grafted nanosilica at 1.9 MPa and 20 °C.

Gas permeation tests using hydrogen were performed to evaluate possible interface improvement (void elimination) by aminosilane grafting to silica nanoparticles. The results of the hydrogen permeation tests through pure cellulose acetate and cellulose acetate-based nanocomposite membranes are shown in Figure 7. The membrane thickness used to calculate the permeability (eq 2) in repeated experiments is presented in Table S1 (Supporting Information). The findings confirm the improved interfacial contact between cellulose acetate and grafted nanosilica compared to that of pristine nanosilica. A reduction in gas permeability of 93% for the grafted nanosilica membrane (9.6 Barrer) was found compared to the membrane containing pristine nanosilica (138.9 Barrer). Moreover, the silanization of the filler apparently reduces the degree of anisotropy within the membrane by improving the distribution of the nanoparticles within the polymeric phase. This is indicated by a high standard deviation for the pristine nanosilica membrane, while the obtained permeation values for two grafted nanosilica membranes were almost identical in the repeated experiments (Figure 7). The observed hydrogen permeability through pure cellulose acetate membrane (13.3 Barrer) implies that along with the absence of voids, the main effect of the grafted nanoparticle addition is decreasing the volume between polymeric chains, thereby contributing to lower hydrogen permeation.

Limited research has been reported on fabricating cellulose acetate-based composite membrane gas separation. The incorporation of NaY-type zeolite,^41^ MWCNTs,^42^ NH_2_-MIL-53(Al) crystals,^43^ and pristine silica particles prepared via the sol–gel method^19^ into cellulose acetate has been investigated. Najafi et al.^19^ synthesized silica nanoparticles (no information was given on the particle size and porosity) and investigated them as fillers for cellulose acetate-based composites in the permeation of pure gases (CO_2_, N_2_, and O_2_) at 1 MPa and 25 °C. The authors reported an increase in the permeability of CO_2_ from 6.32 to 7.3 Barrer, and a reduction in the permeability of N_2_ from 0.18 to 0.09 Barrer with the increment in silica content of the composite membranes up to 20 wt %. Hamidavi et al.^44^ reported hydrogen permeability through poly(ether imide)/hydrophobic silica nanocomposite membrane of 0.4 Barrer at 0.6 MPa and 25 °C. Commercial hydrophobic fumed silica (SiO_2_, AEROSIL R974, 12 nm, BET = 170 ± 20 m^2^/g) was used for the study.^44^ To the best of our knowledge, there are no data available on hydrogen permeability through cellulose acetate nanocomposites. At the same time, most studies consider pressures below 1 MPa, and data on the application of nanocomposite membranes under high pressures are scarce. Our results show a H_2_ permeability of 9.6 Barrer through the cellulose nanocomposite membrane with silanized commercial silica nanoparticles at an elevated pressure of 1.9 MPa and a reduction in permeability compared to pure polymeric membranes (13.3 Barrer). Such a “reinforcement” of the polymeric membrane makes this composite a candidate for further studies in the separation of gas mixtures containing CO_2_. Namely, the presence of CO_2_ at elevated pressures leads to polymer plasticization, which is supposed to be suppressed by adding nanoparticles.^43^

The presence of CO_2_ under elevated pressures leads to polymer swelling and might contribute to its plasticization. Consequently, it may also affect the interphase in the composite membranes. The literature lacks data on composite aging due to prolonged exposure to CO_2_ under elevated pressures, which will be an aim of our research in the future. Therefore, in this study, we report the results of the CO_2_ permeation tests as preliminary data. Since the H_2_ permeation tests revealed the existence of interfacial voids in the membrane with pristine nanosilica, this membrane was excluded from further study. The membrane with silanized nanosilica and neat cellulose acetate membrane were considered for CO_2_ permeation at 20 °C and 1.9 MPa. The measurements show a higher permeability of CO_2_ through the membrane with grafted nanosilica (21.7 Barrer) compared to that of the neat polymeric CA membrane (16.8 Barrer). Compared to H_2_, both membranes show a higher CO_2_ permeability. The enhanced permeability of the CA membrane is attributed to the higher solubility of CO_2_ in the polymer matrix in comparison to H_2_, while in the membrane with modified nanosilica, the permeability is additionally enhanced by a strong affinity of the amino group toward the CO_2_, which ultimately results in a higher CO_2_ solubility. Furthermore, the enhancement of CO_2_ permeability due to the presence of amino groups in the membrane with modified nanosilica can be accredited to the interaction between the amino group and CO_2_ molecules through a carbamate zwitterion mechanism, resulting in a high CO_2_ solubility.^12,45−47^ All in all, besides interfacial contact improvement and void elimination as evidenced by H_2_ permeation, preliminary permeation experiments with CO_2_ indicate an increase in CO_2_/H_2_ selectivity from 1.26 (pure polymeric membrane) to 2.26 (composite membrane with grafted nanosilica) at the investigated experimental conditions.

### 3.3 Starch-Chitosan Blend Composite Membranes

Composite membranes were prepared with 15 and 20 wt % of silanized and pristine nanosilica. Similarly, like in cellulose acetate-composites, the silanization allowed for a more uniform distribution of less agglomerated filler, as evidenced in Figures S12 and S13 (Supporting Information). The surface of composites with the silanized filler was considerably smoother, and the cross-sections revealed less agglomerated filler than when pristine nanosilica was used. SEM images presenting the compact structure of SC blends without a filler are shown in Figure S14 (Supporting Information).

#### 3.3.1 Thermal Properties of Starch-Chitosan Blend Composite Membranes

The thermal properties of SC membranes functionalized with pristine and silanized nanosilica are shown in Table 3. DSC thermograms are presented in Figure S15 (Supporting Information). The SC membrane without fillers showed two well-defined endothermic peaks at 107 and 258 °C related to the melting and phase inversion of the crystalline phases of starch and chitosan, respectively. This observation could be due to the incomplete miscibility of starch and chitosan in blends obtained by the solvent-casting method.^48^

**Table 3:** Thermal Parameters (DSC and TGA Analyses) of the Starch-Chitosan (SC) Membranes Functionalized with Pristine Nanosilica (NS) and Silanized Nanosilica (sNS)

sample	*T*_m1_ (°C)	Δ*H*_m1_ (J g^–1^)	*T*_m2_ (°C)	Δ*H*_m2_ (J g^–1^)	*T*_o1_ (°C)	*T*_o2_ (°C)	*T*_d1_ (°C)	*T*_d2_ (°C)
control SC	107 ± 2^a^	257 ± 44	258 ± 4	163 ± 15	184 ± 2	245 ± 1	215 ± 1	291 ± 1
SC-15% NS	110 ± 5^a^	192 ± 34	265 ± 2	117 ± 10	188 ± 9	255 ± 2	223 ± 9	291 ± 0
SC-20% NS	110 ± 3^a^	181 ± 22	263 ± 2	116 ± 4	191 ± 4	257 ± 1	225 ± 6	291 ± 1
SC-15% sNS	103 ± 1^a^	148 ± 10	262 ± 2	115 ± 1	179 ± 4	257 ± 1	214 ± 4	290 ± 1
SC-20% sNS	105 ± 1^a^	149 ± 12	262 ± 0	114 ± 1	178 ± 5	257 ± 1	213 ± 6	289 ± 1

The melting enthalpy of starch (257 J g^–1^) and chitosan (163 J g^–1^) phases decreased up to 30% (181 J g^–1^) and 29% (116 J g^–1^), respectively, due to the addition of pristine nanosilica without significant differences between the samples in terms of the filler content (15 and 20 wt %), showing that nanosilica had the ability to limit the crystal phase growth of both starch and chitosan during membrane formation, increasing the formation of amorphous regions in the nanoreinforced composites. This phenomenon has been particularly associated with an interplay occurring via hydrogen bonding between the –OH groups of starch^49^ and –NH and −OH of chitosan^50^ with –OH groups of nanosilica, which reduced the chance of hydrogen bonding formation between the polymeric chains of both starch and chitosan, consequently reducing crystallinity.

On the other hand, the addition of silanized nanosilica decreased the melting enthalpy of the starch matrix to a greater extent (up to 42%) without differences between the different filler contents. This might be because silanization made the nanosilica more evenly distributed, which, in turn, made it easier for the modified filler to interact via amino groups of grafted aminosilane molecules with the starch polymeric matrix. The silanization of nanosilica did not decrease the phase inversion enthalpy of chitosan.

The results of the TGA are presented in Table 3 and Figure S16 (Supporting Information). The SC membrane, as well as the SC composite membranes with pristine and silanized nanosilica, exhibited three degradation stages, corresponding to the evaporation of free and bound water in the membrane, the degradation of the starch matrix, and the degradation of the chitosan matrix, respectively. The addition of pristine nanosilica increased the thermal stability of the starch matrix, increasing the mean values of *T*_o1_ and *T*_d1_. Particularly, the addition of nanosilica at 20 wt % increased the *T*_o1_ of the starch matrix from 184 to 191 °C. Meanwhile, *T*_d1_ of the starch matrix was increased from 215 to 225 °C. This fact agrees with the increase in the *T*_m1_ of the starch matrix from 107 to 110 °C due to the nanosilica addition (Table 3). This thermally protective effect could be attributed to the interaction between nanosilica and the starch matrix via hydrogen bonding.^49,51^ The interaction via hydrogen bonding between nanosilica and the chitosan matrix can also be associated with the increase in the *T*_o2_ of the chitosan matrix from 245 to 257 °C and the *T*_m2_ increase from 258 to 263 °C due to the addition of NS at 20 wt % (Table 3). The addition of silanized nanosilica decreased the thermal stability of the starch phase, decreasing its *T*_o1_ value from 184 to 178 °C and its *T*_d1_ value from 215 to 213 °C without differences between the different filler contents. This fact completely agrees with the decrease in the *T*_m1_ and Δ*H*_m1_ values of the starch phase due to the addition of a grafted filler (Table 3). As was expected due to the unchanged phase inversion enthalpy of the chitosan matrix, the films with silanized nanosilica showed thermal stability very similar to those with the pristine nanoparticles.

#### 3.3.2 Oxygen Permeability of Starch-Chitosan Blend Composite Membranes

The OP values at 23 °C and 0% RH of the SC membrane (control) and SC composite membranes with pristine and silanized nanosilica are shown in Table 4. The control SC membrane (50 wt % chitosan) presented an OP value (0.406 cm^3^ mm/(m^2^ atm day)) similar to the OP value reported by Bonilla et al. for wheat starch-cast films with the same chitosan content (0.08 cm^3^ mm/(m^2^ atm day)).^52^ The OP of the composite SC membrane with pristine nanosilica was increased due to the addition of 15 wt % nanoparticles up to 277% (1.532 cm^3^ mm/(m^2^ atm day)). Such a considerable decrease in OP resistance could be attributed to the large agglomeration of pristine nanosilica inside the polymeric structure, the poor compatibility of the organic and inorganic phases, and the decrease in crystallinity of the nanocomposite (as shown in Table 3). A decreasing crystallinity and particle agglomeration were also related to an increasing OP in PLA films due to the addition of pristine nanosilica at 1 wt %.^53^

**Table 4:** OP of the SC Membranes Functionalized with Pristine NS and sNS

sample	oxygen permeability (cm^3^ mm/(m^2^ atm day))
control SC	0.406 ± 0.123
SC-15% NS	1.532 ± 0.325
SC-20% NS	0.927 ± 0.243
SC-15% sNS	0.016 ± 0.005
SC-20% sNS	0.007 ± 0.001

Table 4 shows that the addition of silanized nanosilica to the SC blend improved its barrier properties against oxygen. Particularly the addition of the filler at 20 wt % decreased the OP by 98.3% compared to the pure SC membrane (from 0.406 cm^3^ mm/(m^2^ atm day) to 0.007 cm^3^ mm/(m^2^ atm day)). The OP value of the composite membrane with silanized nanosilica was significantly lower compared to the membrane with pristine filler. These results could be attributed to the enhanced bounding at the interface due to better compatibility of the silanized filler with the organic matrix and a more homogeneous membrane structure. At the same time, improving the dispersion of silanized nanosilica inside the polymer promotes its oxygen scavenger activity at low humidity levels through the physisorption mechanism.^54^ The presented results show that better compatibility of phases, better filler dispersion, and improvement of silanized nanosilica scavenger activity prevailed over the negative effect of the decrease in the crystallinity of the membranes with the modified filler. Ortenzi et al.^55^ reported a crystallinity increase and improvement of oxygen barrier properties of PLA membranes synthesized via “in situ” polymerization of l-lactide by adding nanosilica or montmorillonite (1.0 wt %), whereby the silanization of the inorganic phase yielded in a larger crystallinity increase and OP decrease of the produced nanocomposites. The lowest reported OP observed was 2.03 cm^3^ mm/(m^2^ atm day).^55^ In this study, we report a considerably lower value of 0.007 cm^3^ mm/(m^2^ atm day), i.e., even further improved membrane performance.

## 4 Conclusions

The feasibility of commercial nanosilica silanization in scCO_2_ and the potential of the modified nanoparticles to be used in composite membrane design for gas separation and packaging were demonstrated. The environmentally friendly grafting of tightly packed nanosilica allowed for minimizing the loss of nanoparticles and the consumption of solvents. The optimal parameters for the reaction at 50 °C were found to be a pressure of 20 MPa and a contact time of 12 h, which yielded a GD of 18.7%. SEM/FIB imaging confirmed a more homogeneous membrane structure with less prominent agglomeration in composite membranes with silanized nanosilica.

The silanization contributed to the improvement of nanosilica compatibility with tested polymers, even at high filler concentrations of up to 20 wt %. The permeability of hydrogen through the cellulose acetate nanocomposite membrane was reduced by 93% when the silanized filler was used. At the same time, CO_2_ sorption isotherms showed reduced sorption potential of the modified filler, suggesting that the resistance to hydrogen permeation by homogeneously distributed filler and the absence of voids is not jeopardized by a potentially competing transport mechanism by adsorption–diffusion through the nanopores. The homogeneous structure of the nanocomposite membrane with the modified filler was also reflected by the high repeatability of the permeation experiments. Bearing in mind that interfacial void elimination and uniform filler distribution are prerequisites for gas-separation composite membrane design, the results indicate that scCO_2_-functionalized nanosilica is a promising filler for that purpose. Our study implies an increase in the CO_2_/H_2_ selectivity of the MMM with grafted nanosilica compared to the pure cellulose acetate membrane.

The modified nanosilica was also shown as a promising filler for the design of packaging with high oxygen barrier properties. The oxygen barrier properties of the SC blend were improved by 98.3% by the modified nanosilica addition at 20 wt %, yielding a membrane with an extremely low OP value of 0.007 cm^3^ mm/(m^2^ atm day), similar to those reported for metalized polymeric films. The phenomenon can be explained by a well-dispersed filler, as well as the improved compatibility of the grafted inorganic and organic phases, which subsequently resulted in the absence of interfacial voids.

## Supporting Information Available

Additional experimental details, materials, and methods, including photographs of the experimental setup (PDF)
