# Supplementary material for: Enhanced Performance of Nanocomposite Membranes by an Environmentally Friendly High-Pressure Silanization Method
Source: ACS Omega. 2025 Mar 3;10(9):9484–95. doi: 10.1021/acsomega.4c10503 (PMC11904675; doi:10.1021/acsomega.4c10503)
Supplement: Supplementary file 1 — ao4c10503_si_001.pdf [file ao4c10503_si_001.pdf]

Supporting Information

Enhanced performance of nanocomposite membranes by an environmentally friendly high-pressure silanization method

Patience Nnenna Abugu[†,‡], *Hanin Samara*[ǂ,*], *Adrián Rojas*[⸸,⸷], *Ewelina Ksepko*[†], *Mariusz Nowak*[†], *Ximena Valenzuela*[⸸,⸷], *Marcin Tyrka*[†], *Philip Jaeger*[ǂ], *Irena Zizovic*[†,*]

[†] Faculty of Chemistry, Wroclaw University of Science and Technology, Wyb. Wyspianskiego 27, 50-370 Wroclaw, Poland

[‡] Department of Materials Science and Metallurgy, University of Cambridge, 27 Charles Babbage Road, CB3 0FS, Cambridge, United Kingdom

[ǂ] Institute of Subsurface Energy Systems, Clausthal University of Technology, Agricolastr. 10, 38678 Clausthal-Zellerfeld, Germany

[⸸] Packaging Innovation Center (LABEN), Department of Science and Food Technology, Faculty of Technology, University of Santiago of Chile (USACH), Obispo Umaña 050, Santiago 9170201, Chile

[⸷] Center for the Development of Nanoscience and Nanotechnology (CEDENNA), Santiago 9170124, Chile

Corresponding authors: irena.zizovic@pwr.edu.pl, hanin.samara@tu-clausthal.de

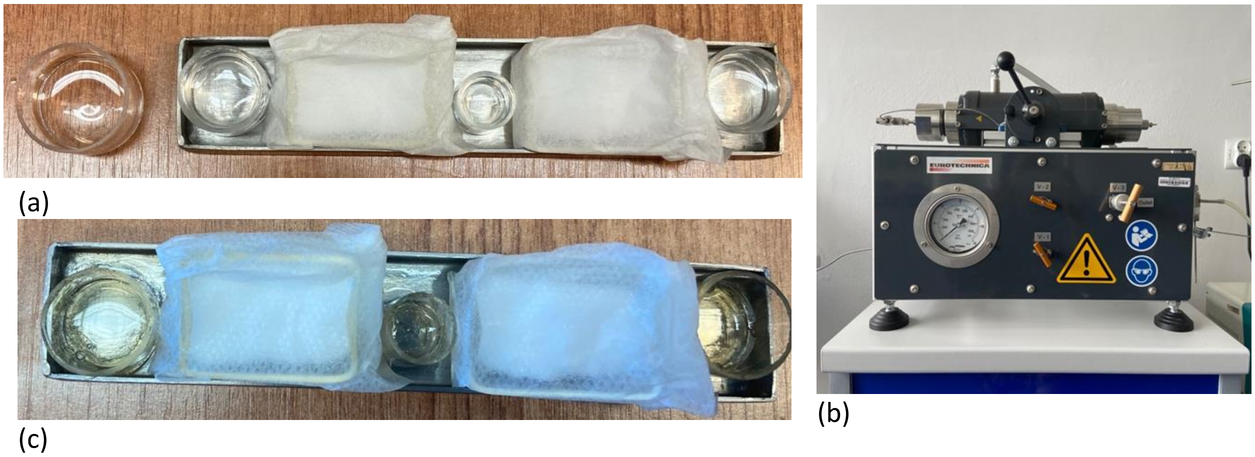

Figure S1. The spatial arrangement of APDEMS solution (round glass containers) and nanosilica (glass containers wrapped by filter paper) (a), which was placed in a tilting high-pressure vessel put in the horizontal position (b); the tray with the containers after the silanization reaction in $scCO_2$ at 50°C and 20MPa for 12 h (c).

S-2.4. Materials characterization

Fourier transform infrared (FTIR) spectroscopy was employed to pristine and grafted silica nanoparticles to verify the silanization reaction. The spectra were recorded in ATR mode using a Nicolet iS50 spectrometer (Thermo Fisher SCIENTIFIC, Waltham, MA, USA) with a resolution of 4 cm $^{-1}$ at wavenumbers in the range of 500 to 4000 cm^{-1}.

The structural properties of the composite membranes were investigated by a two-beam microscope SEM/Ga-FIB FEI Helios NanoLab TM 600i (FEI, Thermo Fisher Scientific, Eindhoven, The Netherlands). The energy-focused beam of gallium ions was used to make sample cross-sections of the material. The samples were coated with gold before the analyses.

Thermogravimetric analysis was employed to determine the grafting degree in modified nanoparticles using a thermal analyzer STA 449 F5 Jupiter coupled to a quadrupole mass spectrometer QMS 403 Aëolos Quadro (Netzsch, Germany). A 10 mg sample was placed in an alumina crucible, heated from room temperature in a nitrogen atmosphere (100 mL/min) with a heating rate of 10°C/min to the desired temperature of 120°C, and kept at this temperature for 10 min and then heated to 800°C with a heating rate of 20°C/min. After reaching 800°C, the sample was maintained at this temperature for 10 min.

Sorption of CO_2 at 273 K using Micromeritics Accelerated Surface Area and Porosimetry System (ASAP) 2020 was used to investigate nanopowder surface areas before and after silanization. The primary outgassing was performed at 240 °C and at a pressure below 1 μm Hg for three hours. The secondary outgassing (240 °C, < 1 μm Hg) lasted one hour. The pressure ratio (p/p_0) during the analyses ranged from 0 to 0.96.

A Mettler-Toledo model STAR 822e (Schwerzenbach, Switzerland) device coupled with a HAAKE EK 90/MT (Newington, USA) cooling system was used for the differential scanning calorimetry (DSC) testing of starch-chitosan blend membranes. Under a nitrogen environment, a single heating of 4–7 mg of material was conducted from 0 to 360 °C at a constant rate of 10

°C/min. To perform thermogravimetric analysis (TGA) of starch-chitosan blend membranes, a Mettler Toledo Gas Controller GC20 Stare System TGA/DCS (Schwerzenbach, Switzerland) was utilized. 7 mg of the sample was added to a porcelain capsule and heated from 30 °C to 700 °C (flow rate 50 mL/min) at 10 °C/min in a nitrogen atmosphere. The parameters obtained include the temperature of degradation at 2.5% of weight loss (T_o) and the temperature of maximal deterioration (T_d).

The oxygen permeability of starch-chitosan blend membranes was tested using a Mocon Ox-TRANR 2/20 (Modern Controls Inc., Minneapolis, Minn.) fitted with a coulometric oxygen sensor at 23 °C and 0% RH. The tests were conducted until a steady-state oxygen transmission rate was reached, in compliance with ASTM D3985-81. A desktop computer was linked to the Ox-TRANR 2/20, and DOS software was used to acquire the output values.

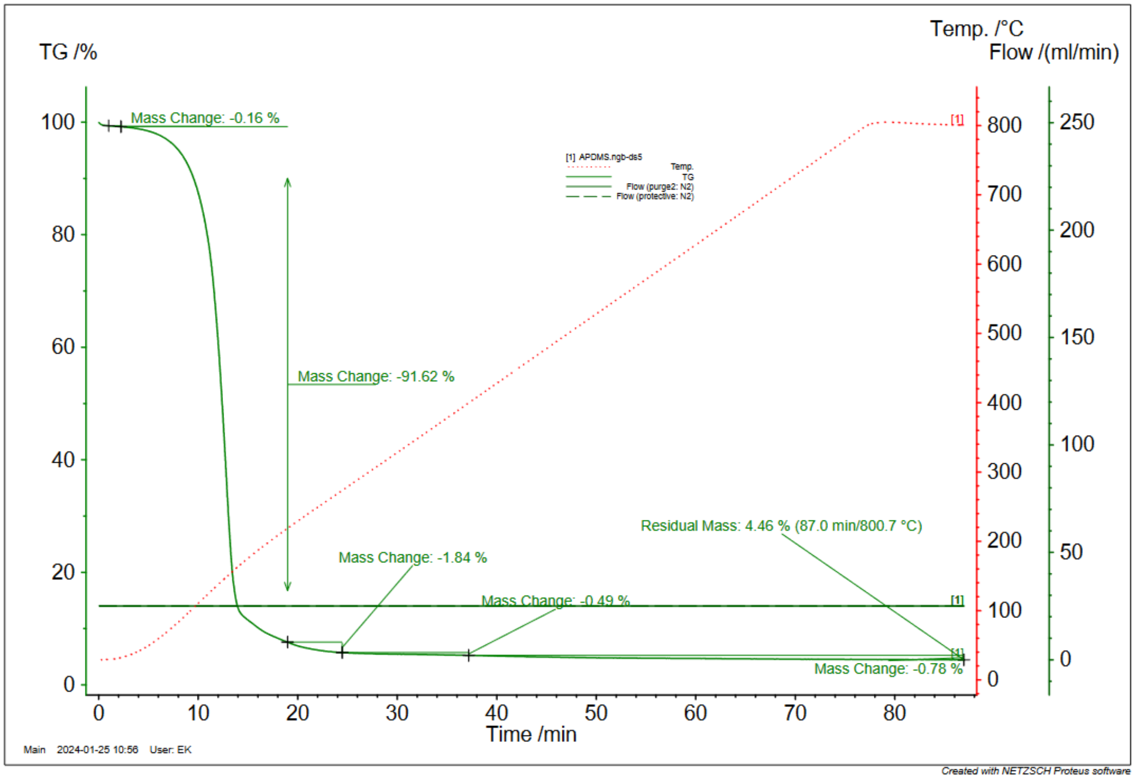

Figure S2. The TGA curve for pure APDEMS.

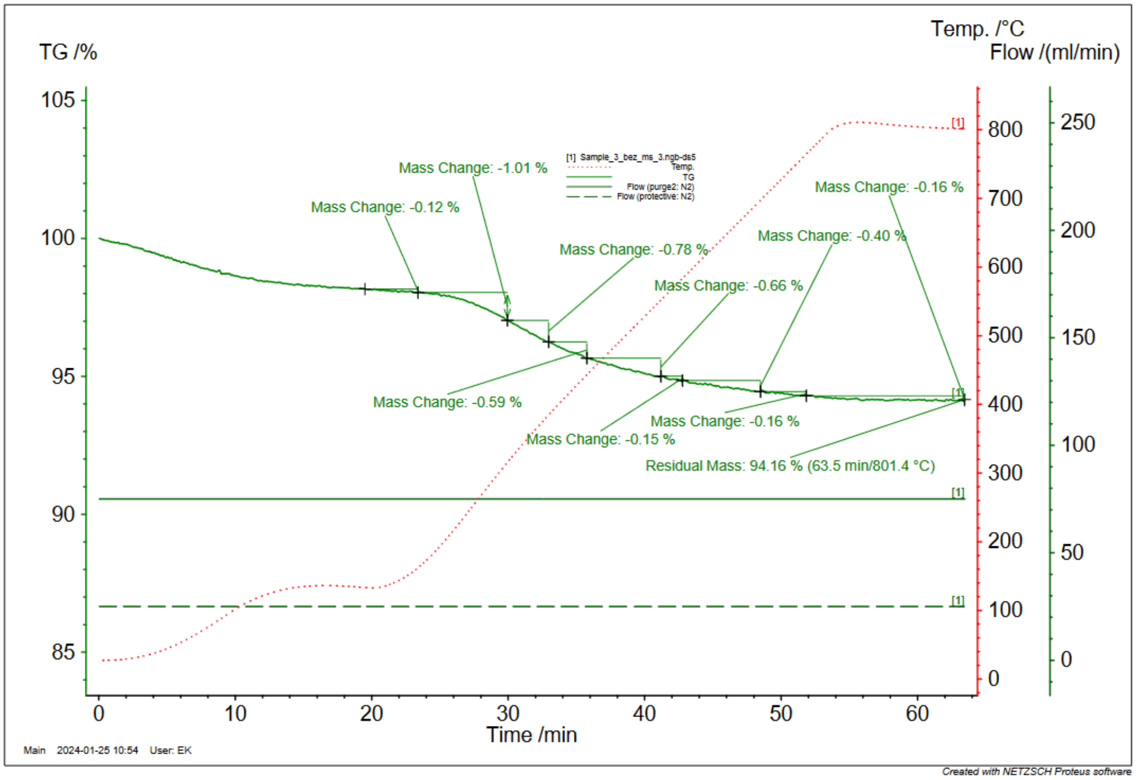

Figure S3. The TGA curve for pristine nanosilica.

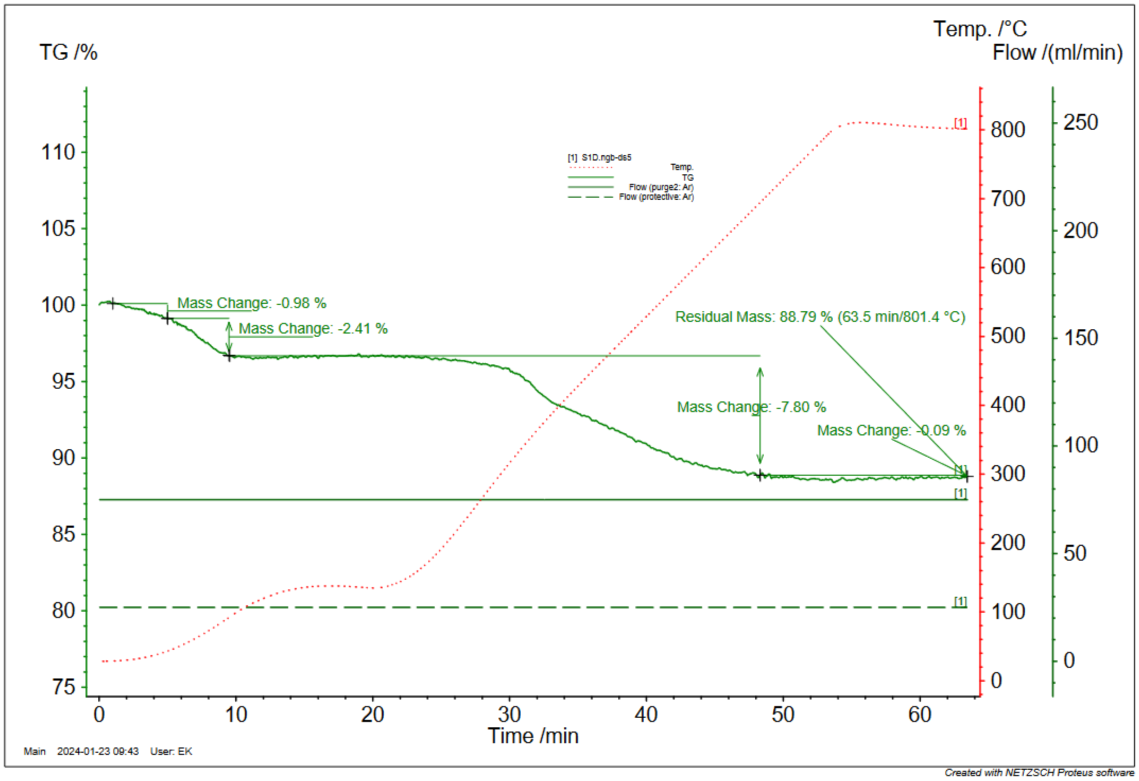

Figure S4. The TGA curve of sample 1 (nanosilica grafted at 20 MPa for 8 h, SSI).

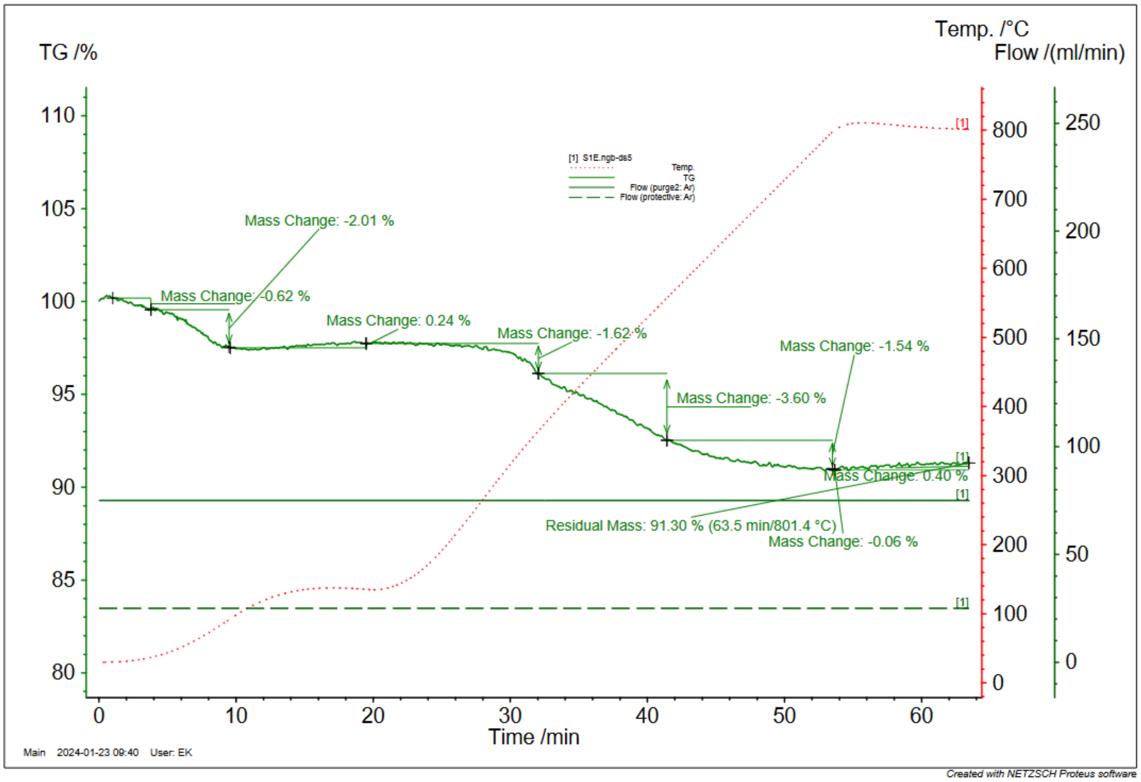

Figure S5. The TGA curve of sample 2 (nanosilica grafted at 25 MPa for 8 h, SSI).

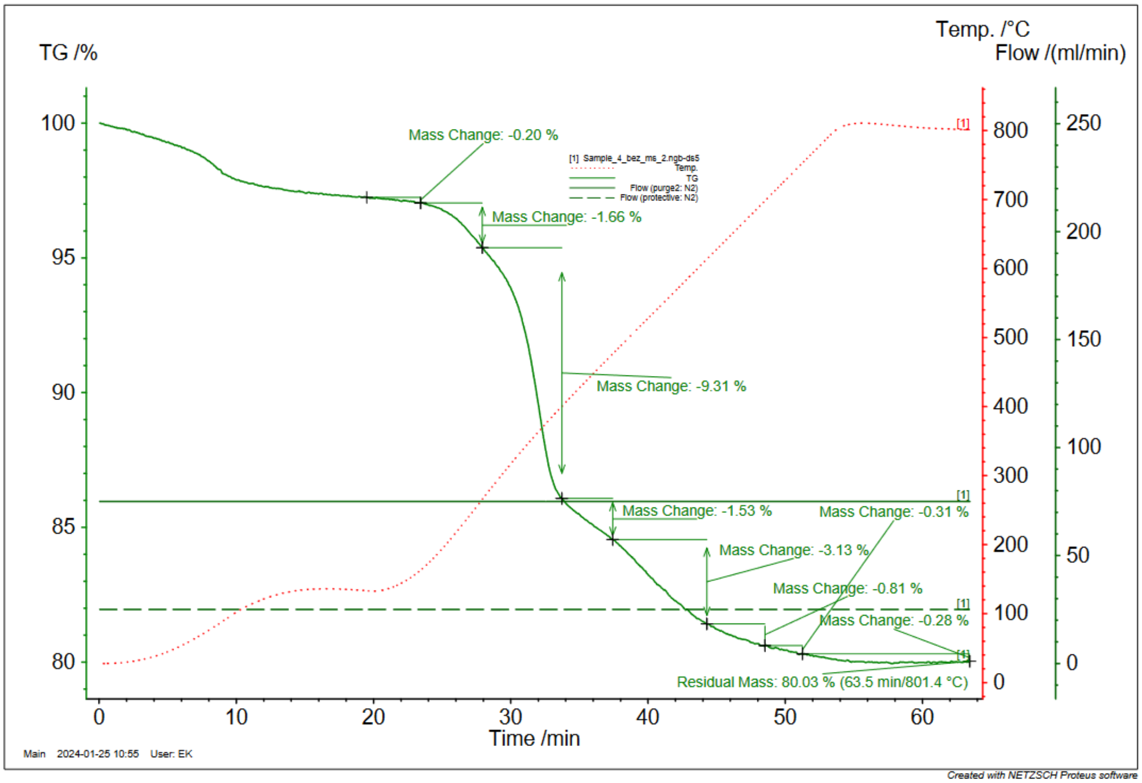

Figure S6. The TGA curve of sample 3 (nanosilica grafted at 20 MPa for 12 h, SSI).

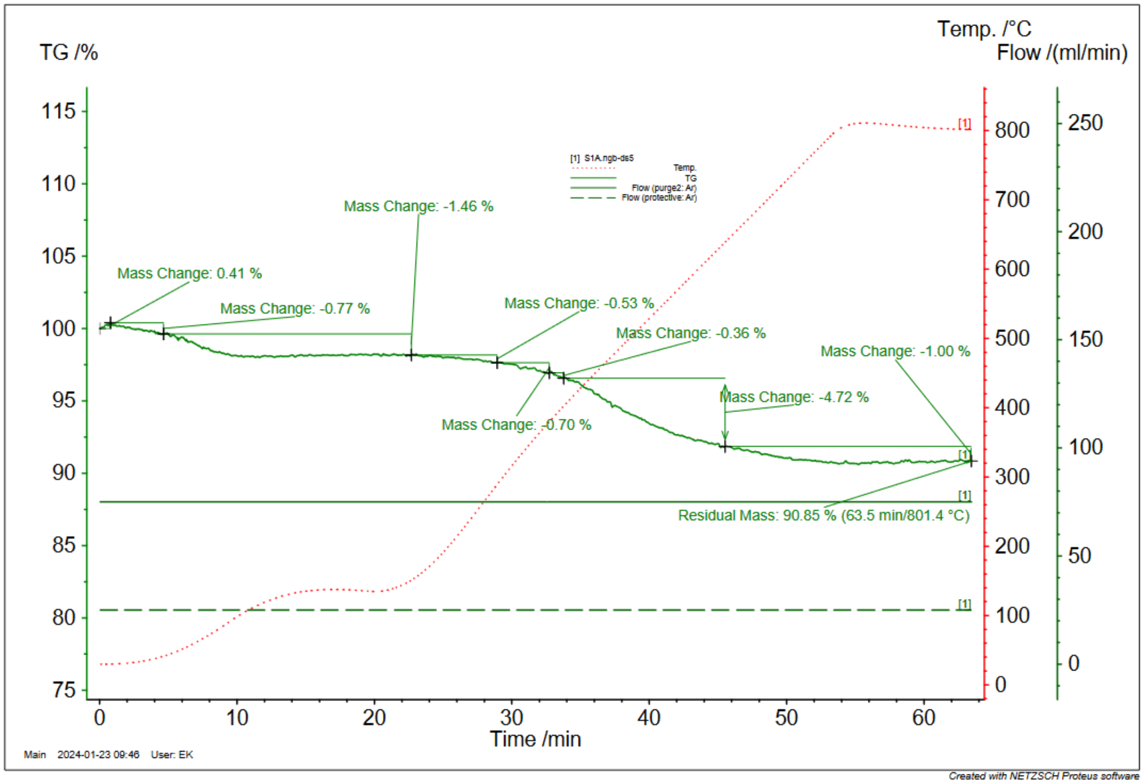

Figure S7. The TGA curve of sample 4 (nanosilica grafted at 12 MPa for 1 h, SAI).

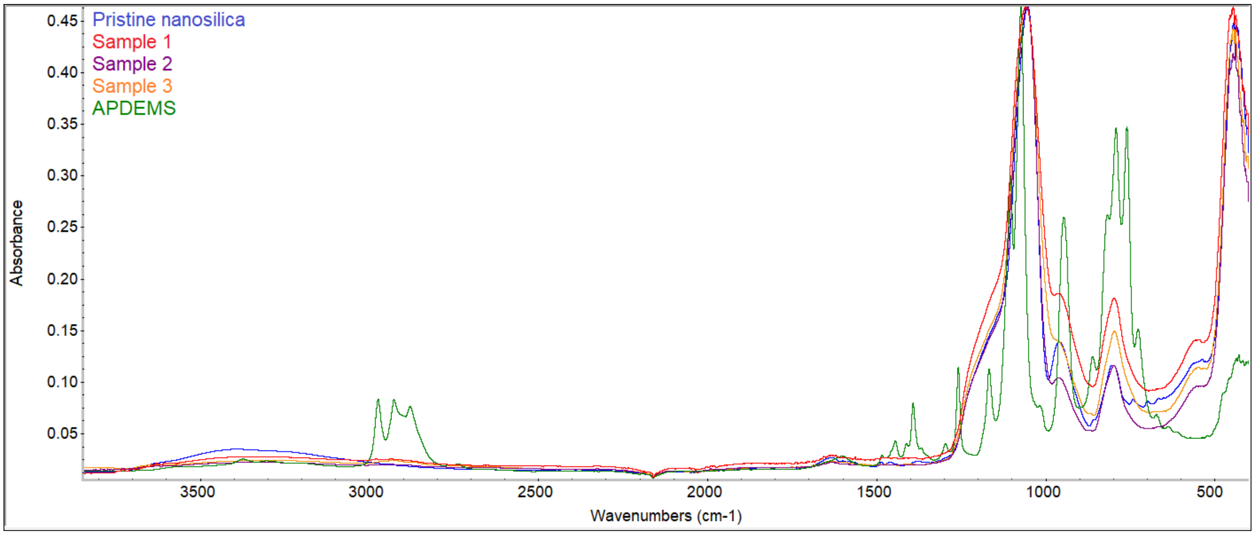

Figure S8. FTIR spectra of pristine and grafted nanosilica, and APDEMS.

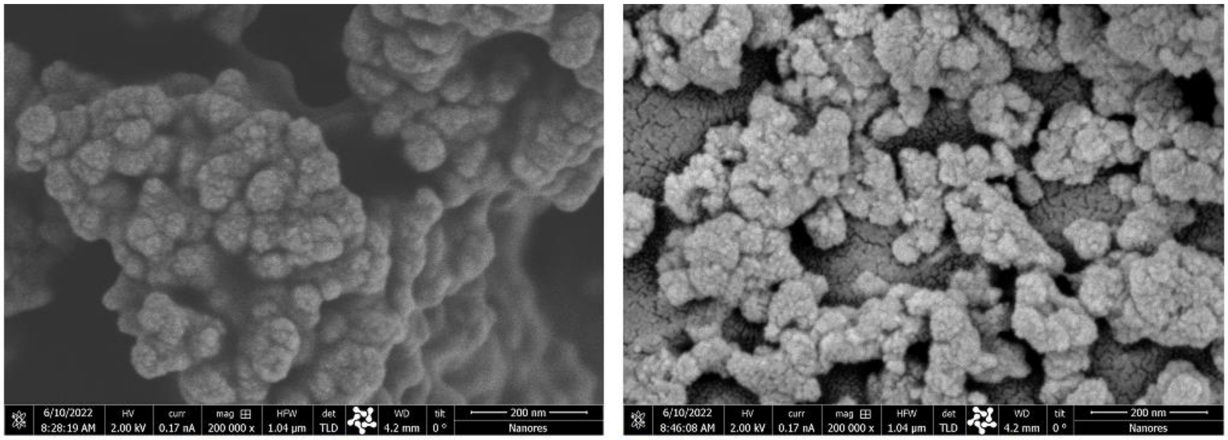

(a) Pristine nanosilica (bar = 200 nm) (b) Grafted nanosilica (bar = 200 nm)

Figure S9. SEM images of pristine and grafted silica nanoparticle powders.

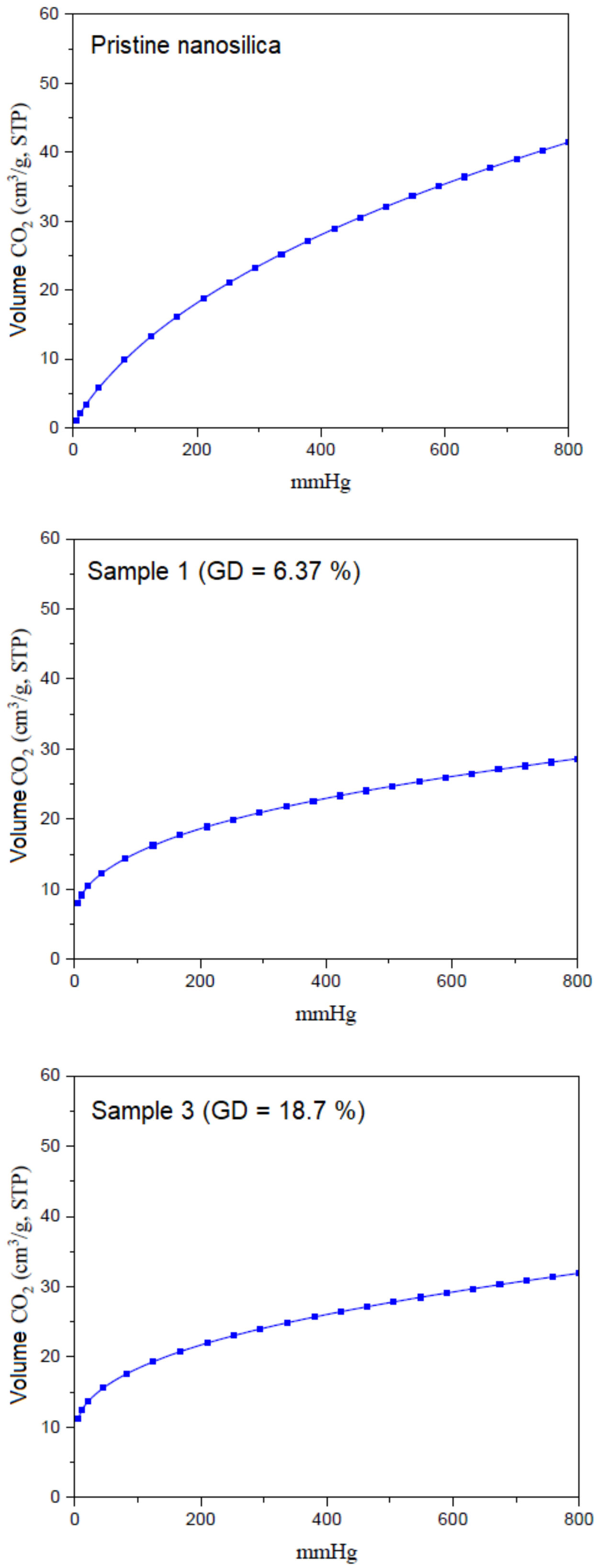

(a)

(b)

(c)

Figure S10. Adsorption isotherms for CO2 at 273 K for pristine nanosilica (a), sample 1 (b), and sample 3 (c).

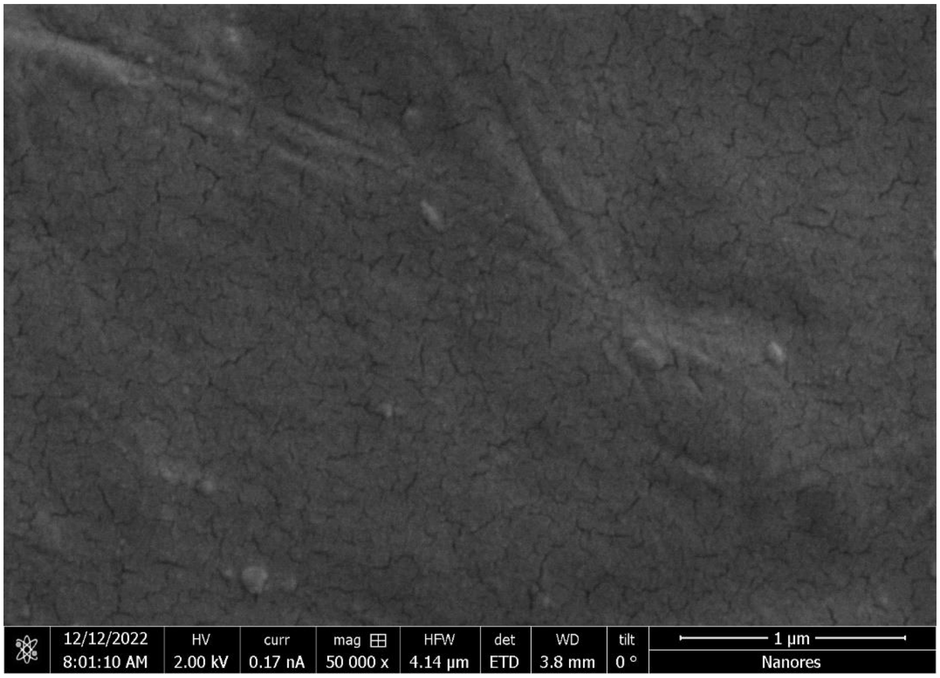

(a)

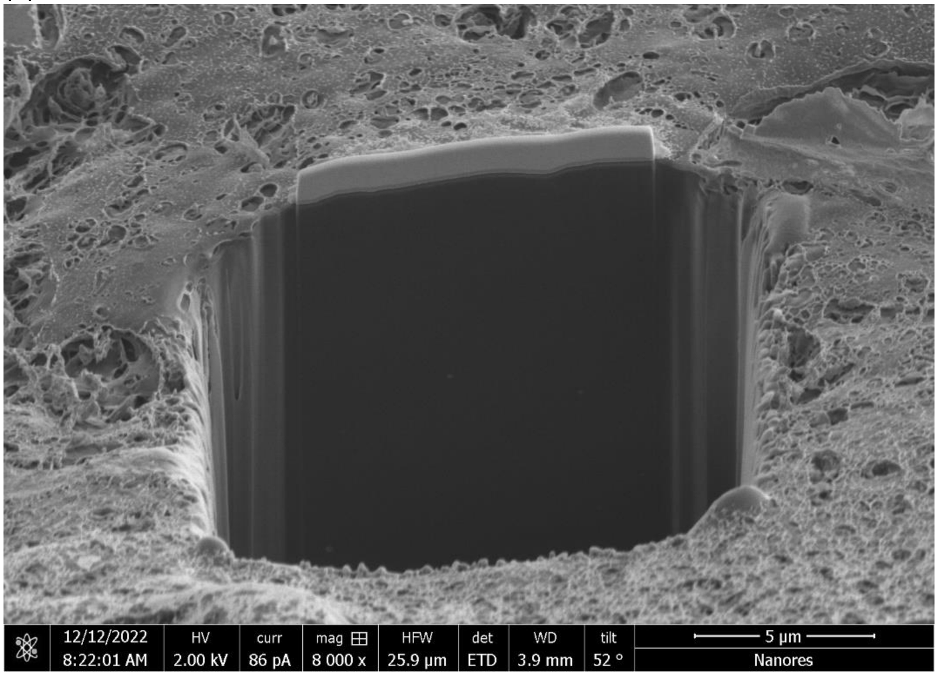

(b)

Figure S11. SEM images of neat cellulose acetate membrane: surface, bar = 1 µm (a); cross-section, bar = 5 µm (b).

Table S1. Cellulose acetate-based membrane thickness used for the permeability calculations

Membrane	Sample number	Gas	Thickness (mm)
Pure cellulose acetate	1	H2	0.14
	2	H2	0.19
Composite with pristine nanosilica	1	H2	0.22
	2	H2	0.22
Composite with silanized nanosilica	1	H2	0.16
	2	H2	0.15
Pure cellulose acetate	1	CO2	0.13
	2	CO2	0.14
Composite with silanized nanosilica	1	CO2	0.12
	2	CO2	0.15

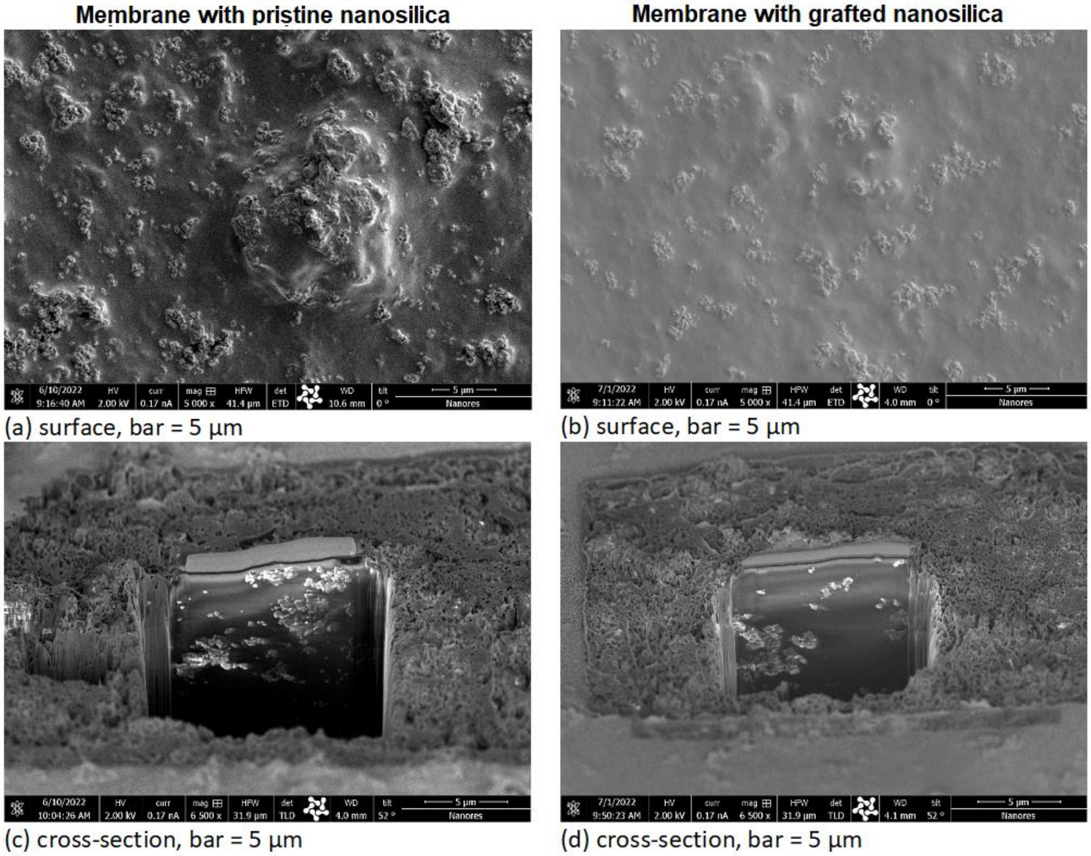

Figure S12. SEM images of surfaces (a, b) and cross-sections (c, d) of starch-chitosan composite membranes with 15 % wt. pristine and grafted nanosilica.

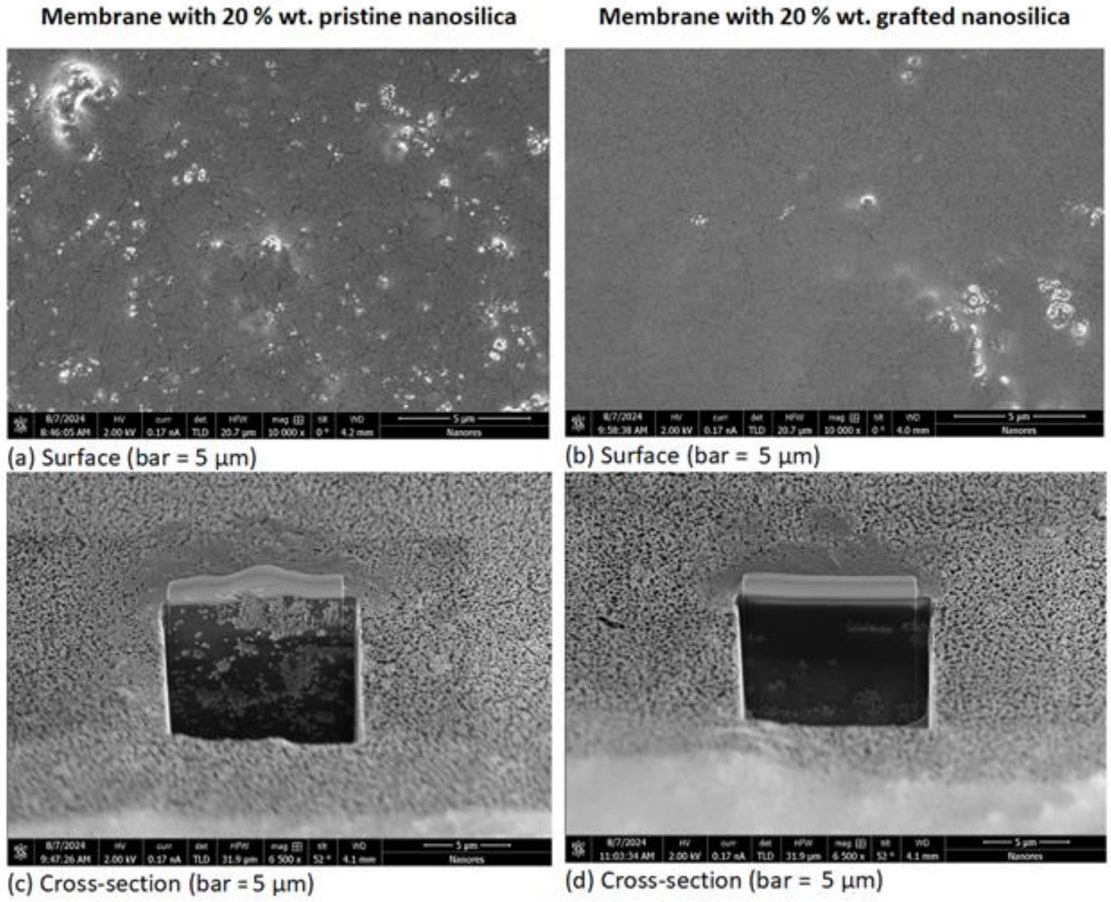

Figure S13. SEM images of surfaces (a, b) and cross-sections (c, d) of starch-chitosan composite membranes with 20 % wt. pristine and grafted nanosilica.

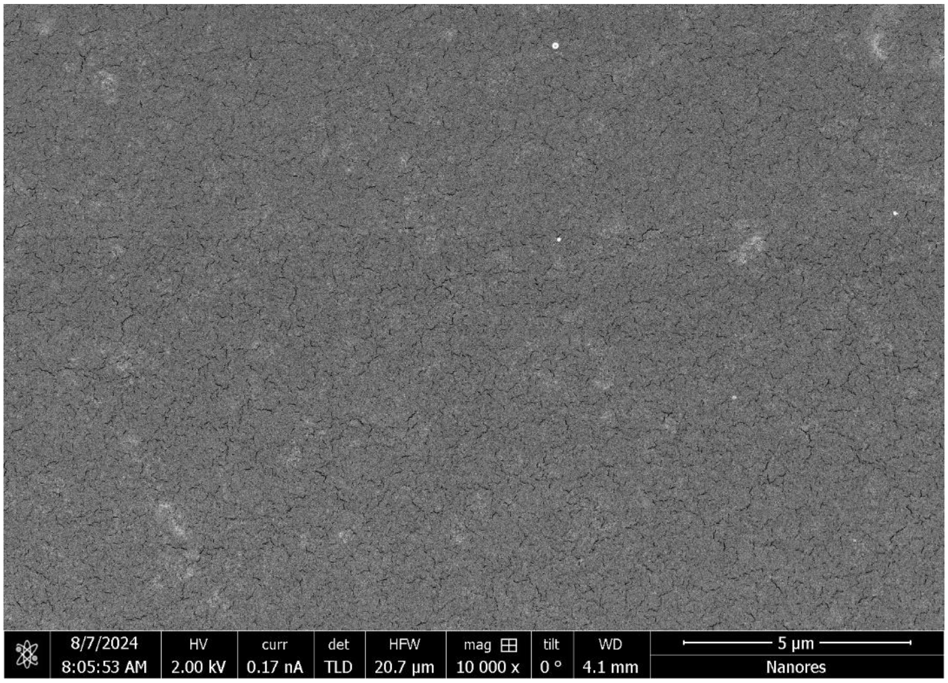

(a)

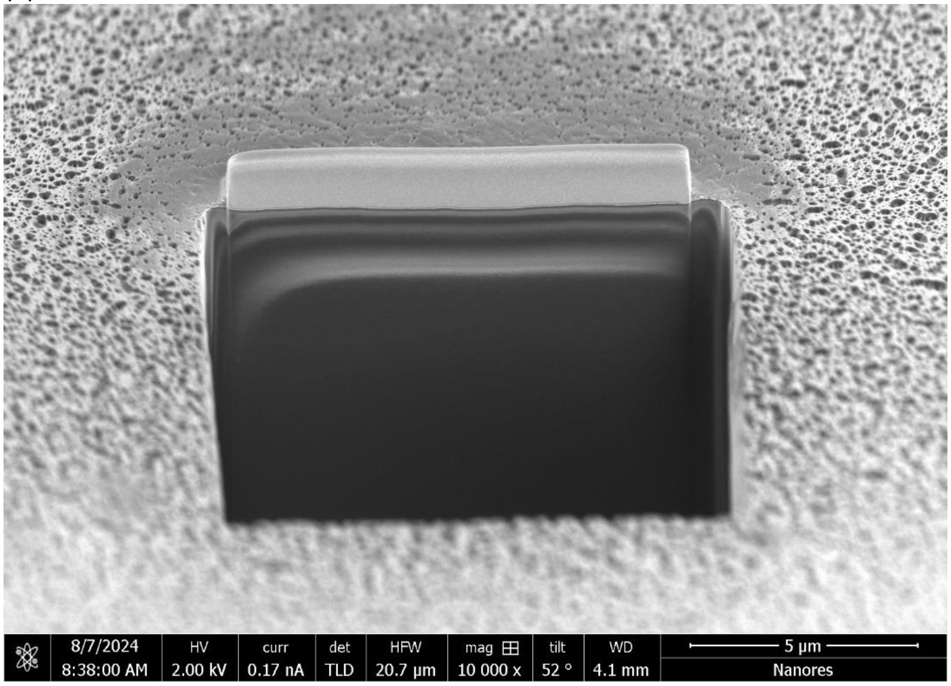

(b)

Figure S14. SEM images of neat cellulose acetate membrane: surface, bar = 5 µm (a); cross-section, bar = 5 µm (b).

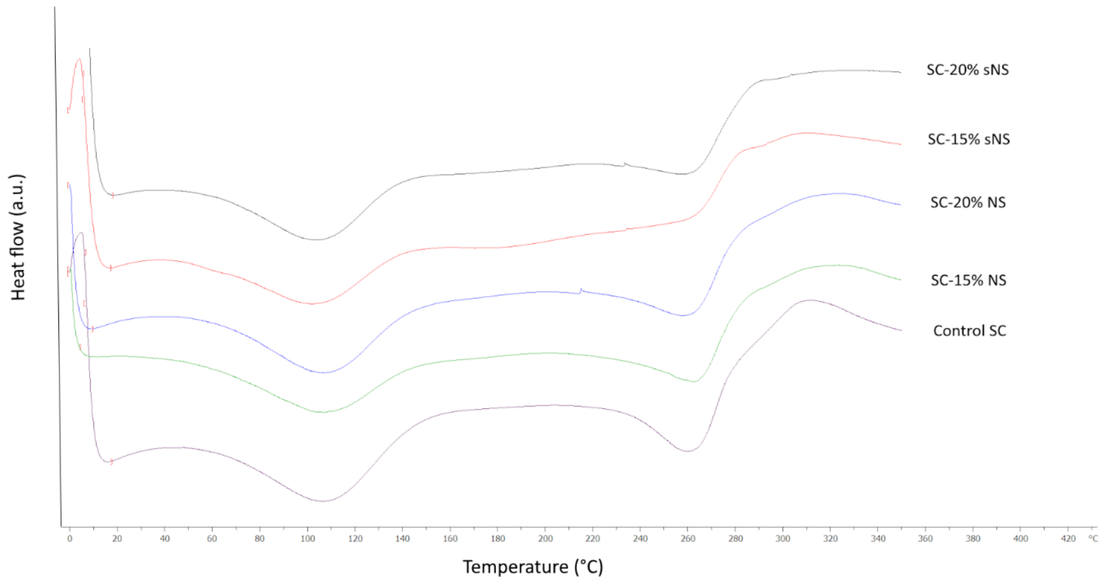

Figure S15. DSC representative thermograms of SC membranes functionalized with nanosilica (NS) and silanized nanosilica (sNS).

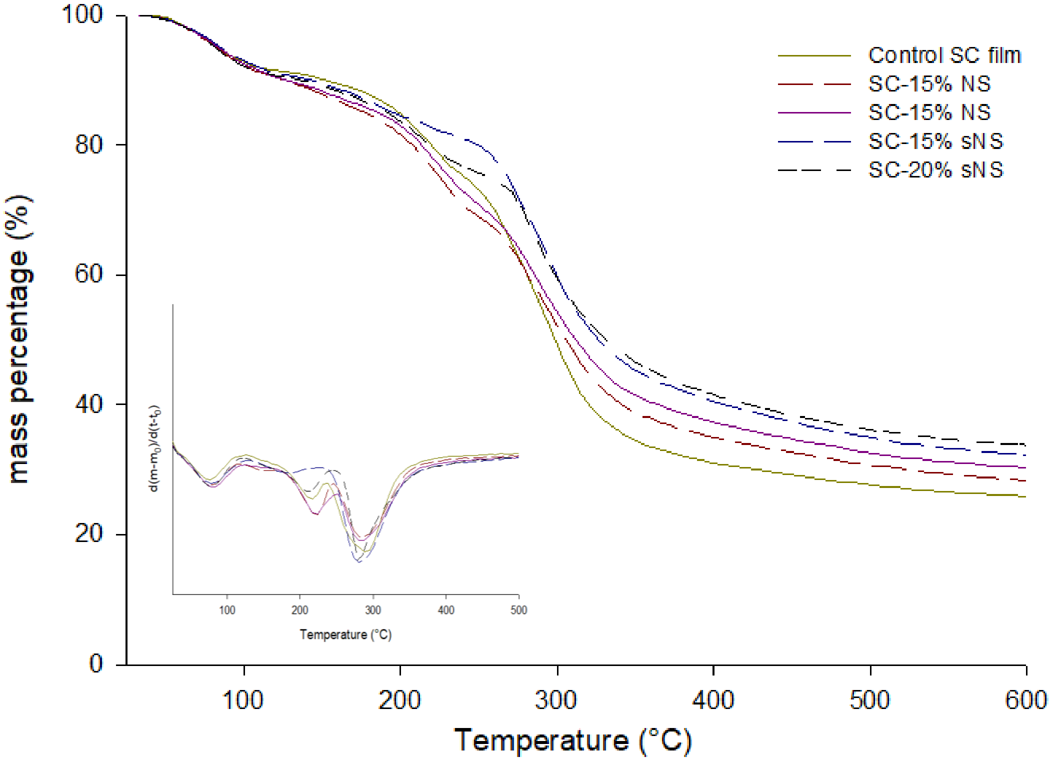

Figure S16. TGA and DTG thermograms of SC membranes functionalized with nanosilica (NS) and silanized nanosilica (sNS).
